# Iturinic Lipopeptide Diversity in the *Bacillus subtilis* Species Group – Important Antifungals for Plant Disease Biocontrol Applications

**DOI:** 10.3389/fmicb.2019.01794

**Published:** 2019-08-07

**Authors:** Christopher A. Dunlap, Michael J. Bowman, Alejandro P. Rooney

**Affiliations:** ^1^Crop Bioprotection Research Unit, National Center for Agricultural Utilization Research, Agricultural Research Service, United States Department of Agriculture, Peoria, IL, United States; ^2^Bioenergy Research Unit, National Center for Agricultural Utilization Research, Agricultural Research Service, United States Department of Agriculture, Peoria, IL, United States

**Keywords:** biocontrol, probiotic, FZB42, QST713, amyloliquefaciens, fengycin, PGPR, biostimulant

## Abstract

Iturins and closely related lipopeptides constitute a family of antifungal compounds known as iturinic lipopeptides that are produced by species in the *Bacillus subtilis* group. The compounds that comprise the family are: iturin, bacillomycin D, bacillomycin F, bacillomycin L, mycosubtilin, and mojavensin. These lipopeptides are prominent in many *Bacillus* strains that have been commercialized as biological control agents against fungal plant pathogens and as plant growth promoters. The compounds are cyclic heptapeptides with a variable length alkyl sidechain, which confers surface activity properties resulting in an affinity for fungal membranes. Above a certain concentration, enough molecules enter the fungal cell membrane to create a pore in the cell wall, which leads to loss of cell contents and cell death. This study identified 330 iturinic lipopeptide clusters in publicly available genomes from the *B. subtilis* species group. The clusters were subsequently assigned into distinguishable types on the basis of their unique amino acid sequences and then verified by HPLC MS/MS analysis. The results show some lipopeptides are only produced by one species, whereas certain others can produce up to three. In addition, four species previously not known to produce iturinic lipopeptides were identified. The distribution of these compounds among the *B. subtilis* group species suggests that they play an important role in their speciation and evolution.

## Introduction

Iturins are an important class of lipopeptides that have been widely studied for their antibiotic activities and are produced by members of the *Bacillus subtilis* group (Ongena and Jacques, 2008). Iturins were first reported as an antibiotic produced by *B. subtilis* in 1950 and named after the Ituri region in the Congo where the strain was isolated (Delcambe, 1950). The chemical structure of iturin A was later reported to be a cyclic heptapeptide with an alkyl chain (Peypoux et al., 1978). The mode of action of these lipopeptides was shown to be pore formation in cell membranes (Besson et al., 1984; Maget-Dana et al., 1985). All of them have been shown to have strong antifungal activity, and they are known active ingredients in many biological control products that target fungal plant pathogens (Ongena and Jacques, 2008). In addition to their antifungal activity, they have been shown to induce defense responses in plants (Farace et al., 2015; Park et al., 2016; Wu et al., 2018).

**FIGURE 1:**
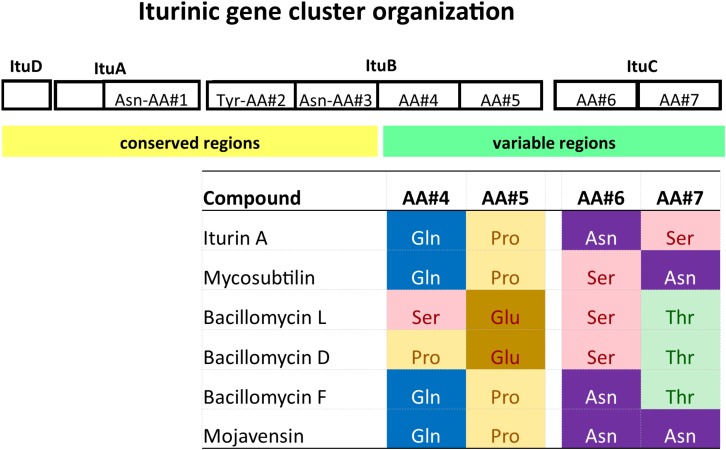
Gene organization and structure of a generic iturinic lipopeptide biosynthesis cluster and sequence of the cyclic heptapeptide of iturinic lipopeptides. The first three positions are Asn-Tyr-Asn for all compounds.

Several other lipopeptides have been identified as being closely related to iturins: mycosubtilin (Peypoux et al., 1976, 1986), bacillomycin L (Besson et al., 1977), bacillomycin D (Peypoux et al., 1980, 1981), bacillomycin F (Mhammedi et al., 1982), and mojavensin A (Ma et al., 2012). Along with iturin, these compounds form the iturinic lipopeptide multigene family. A defining feature of this family is that the first three amino acids of the cyclic heptapeptide are shared among members, whereas the remaining four amino acids are variable (Figure 1). In addition, family members exist as different alkyl isomers and are often produced as a mixture of closely related alkyl tails (e.g., C_14_, iso-C_15_, anteiso-C_15_) (Hourdou et al., 1989; Iwase et al., 2009). In this study, we only considered the nature of the cyclic peptide and disregarded the alkyl chain isomers.

Iturinic lipopeptides are encoded by a cluster of four genes for biosynthesis, which is carried out by non-ribosomal peptide synthetases (NRPS). Iturinic lipopeptide genes are also characterized by modular gene organization, which was first described by Donadio et al. (1991) who defined a module as a “repeated unit” consisting of a specific sequence of nucleotides (or deduced amino acids at the protein level) within one or more open reading frames (i.e., genes) (Donadio et al., 1991). In the case of iturinic lipopeptides, there are 7 modules, each consisting of about 1,000 amino acid sites, that are spread across three genes: 1 in the second gene of the cluster, 4 in the third gene, and 2 in the fourth gene (Duitman et al., 1999; Tsuge et al., 2001; Moyne et al., 2004). Each module contributes a single amino acid that the NRPS enzymatic complex forms into a cyclic peptide consisting of seven amino acids. If an amino acid sequence comparison of all cyclic heptapeptides produced by members of the iturinic lipopeptide family is conducted, it becomes apparent that the first three amino acids of the cyclic heptapeptide are conserved among all family members, whereas the fourth through seventh amino acids may vary (Figure 1). This variation in amino acid sequence is used to assign a lipopeptide clusters to different “types.” The goal of this study is to characterize the diversity of iturin lipopeptide family members produced by the *B. subtilis* species group and identify evolutionary mechanisms associated with their diversification.

In recent years, the taxonomy of the *B. subtilis* species group has been updated and clarified as a result of whole genome sequencing (Dunlap, 2015; Dunlap et al., 2015a, b, 2016b). Prior to the availability of genome sequences, the group was plagued with taxonomic inconsistencies and misidentifications. This was primarily due to the low resolution of 16S rRNA sequencing for this group and the general lack of distinguishing phenotypes (Rooney et al., 2009). A second aspect that impacted research in this area was single dimension, low resolution mass spectroscopy (e.g., MALDI of liquid culture supernatant), which is useful in identifying parent ion masses but not useful in distinguishing between structural isomers. This problem is compounded by a number of common structural variants, such as differences in the alkyl chain length (e.g., C_14_, C_15_, C_16_). These all result in parent ion differences of 1 methylene unit (14.02 Da), which is the same for some common amino acid changes in iturinic peptides (e.g., serine to threonine). The nature of these parent ion molecular weight changes makes it impossible to determine their correct assignment without advanced mass spectroscopy approaches (such as, LC-MS/MS). The combination of inconsistent identification of the producing strains and the ambiguous identification of the compounds has clouded some of the past research literature in this area.

## Materials and Methods

### Bacterial Strains

All publicly available genomes in the *B. subtilis* group were downloaded from GenBank on December 30, 2018 and comprised the data set. Genomes with an unusually high number of contigs or pseudogenes were excluded from the analysis. The taxonomy of the genomes was determined using a 6 gene MLSA approach (Rooney et al., 2009) using BIGSdb software (Jolley and Maiden, 2010). Strains confirmed to belong within the *B. subtilis* group were included in the data set.

### Iturinic Lipopeptide Cluster Mining

The genomes in the data set were BLASTed using genes from known iturinic lipopeptide gene clusters with BIGSdb software (Jolley and Maiden, 2010) in order to identify homologous iturinic lipopeptide gene clusters. Putative gene cluster homologs were sorted into groups based on species assignment and high nucleotide similarity (>94%) across the full alignment length. For a given species, if the gene clusters did not share the full alignment length (e.g., a change in amino acid module results in a 3–4 Kbp nucleotide change), a new cluster group was defined to include these homologs. A cluster groups represent a different lipopeptide within a given species. This resulted in 11 species containing one to three gene cluster types for a total of 15 gene cluster groups. A representative from each species and cluster type was selected to confirm the product of the cluster using MS/MS. A representative strain for each cluster group was obtained, except for two, *Bacillus* genomospecies #1 gene cluster group and *Bacillus siamensis* bacillomycin D group, as strains from these groups were not available in public collections and attempts to obtain them from private laboratories were unsuccessful. However, we are confident in their assignments based on high nucleotide sequence similarity (>90%) to the same verified gene cluster in closely related species. A list of links to representative *ituB* and *ituC* genes by gene cluster type and species is provided in Supplementary Table S1. In addition, the 4′-phosphopantetheinyl transferase (*sfp*) (GenBank#WP_003246659.1) required for NRPS activation (Quadri et al., 1998) was confirmed to be present in each genome with an identified iturinic cluster by protein BLAST.

### Phylogenetic Analysis

A core genome phylogeny was used to show the taxonomic relationships that comprise the *B. subtilis* species group. The core genome determination and subsequent alignments were produced for all the type strains in the group with BIGSdb software (Jolley and Maiden, 2010) and consists of 991 genes. The phylogenetic tree was constructed using MEGA X software (Kumar et al., 2018). The neighbor-joining tree was determined using the Tamura-Nei model (0.40, gamma distributed with invariant sites) based on model testing under MEGA X (Kumar et al., 2018). Measures of bootstrap support for internal branches were obtain from 1,000 pseudoreplicates. A second phylogeny was determined using a 5,395 bp fragment of the *ItuA* gene (6963.12358 of GenBank#AB050629.1) and extracted from all the genomes containing an iturinic cluster. It was processed and analyzed as described above. The final tree was limited to one strain per species to facilitate analyses. Syntenic relationships were determined by manually examining the eight genes located before and after the iturinic lipopeptide gene cluster for at least five random references strains from each species. The average nucleotide identity (ANI) of the genomes was determined using OrthoANI software (Lee et al., 2016) and the nucleotide identity percentages of the partial *ituA* gene were determined by BLAST under BIGSdb software (Jolley and Maiden, 2010).

### Mass Spectroscopy

Strains listed in Supplementary Table S2 as “confirmed with MS/MS” were grown in 5 ml Tryptone-Yeast-Glucose media at 37°C until the late stationary phase (∼96 h). The culture media was centrifuged at 13,000 × *g* for 10 min and the supernatant removed. Mass spectrometry of the supernatant samples (25 μL injections) were collected by LC-MS (Thermo Acela HPLC) through a narrow-bore (2.1 μmm × 150 mm, 3 μm particle size) C18 column (Inertsil, GL Sciences, Inc., Torrance, CA, United States) running a gradient elution of 95% A:5% B (eluent A 18 MΩ water/0.1% formic acid, eluent B 100% methanol/0.1% formic acid) to 5% A:95% B over 65 min at a flow rate of 250 μL/min, followed by a 5 min B washout and 10 min re-equilibration, while maintaining a constant column temperature of 30°C. Electrospray positive mode ionization data were collected with a linear ion trap-Orbitrap mass spectrometer (Thermo LTQ-Orbitrap Discovery) under Xcalibur 2.1 control. Prior to LC-MS*^*n*^* experiments the instrument was tuned and calibrated using the LTQ tune mix. Masses corresponding to iturinic lipopeptides were used to limit the collection of MS^2^ data to only the lipopeptides of interest (Supplementary Figure S1A). Tandem mass spectral data of iturinic peptides was collected using collision-induced dissociation [CID, collision energy (CE) = 25 and 35] in the LTQ and higher-energy collision dissociation (HCD, CE = 35 and 45) in the Orbitrap analyzer, as described in Knight et al. (2018). All tandem mass spectra were manually interpreted for assignment of the lipopeptides.

### Comparison to NRPS Substrate Predictor Software

Proteins annotated as ituA, ituB, and ituC based the genes identified in our initial BLASTing and listed in Supplementary Table S1 were subjected to substrate prediction using NRPSsp software (Prieto et al., 2012).

### Development of Primers for *Bacillus velezensis* Strains

Sequences representing *ituB* gene homologs were extracted from all publicly available *B. velezensis* genomes. These sequences were aligned using CLCbio Genomics Workbench 11.0 (Qiagen Inc., Cambridge, MA, United States) and this alignment was used to identify conserved regions of nucleotides, which were used to design primers. Primers were identified for each gene cluster type (e.g., iturin, bacillomycin D, and bacillomycin L). The forward primer (ituB-fwd, 5′-CACGAACAGACAAAACA-3′) is the same for all three sets and is located on the conserved amino acid (AA) #3 module (Figure 1), while the reverse primers are located on variable AA#4 modules (Figure 1). The reverse primers are ituB-iturin-rev, 5′-TGCGCAAAGCATCGT-3′, ituB-bacD-rev5′-CTTGCGGCGTTTGTG-3′, and ituB-R-bacL5′-GGTCGCTCCTGAATCT-3′. The primers were designed to have an annealing temperature of 55 ± 2°C. PCR testing – DNA was extracted from 1 day old cultures grown on TGY for each isolate using the QIAamp DNA Mini QIAcube Kit (Qiagen Inc., Germantown, MD, United States) according to manufacturer’s protocol instructions. PCR reactions were carried out in 25 μl volumes using 2× Amplitaq gold master mix, 50 ng of DNA and 1 μM primers (Bvel-f and Bvel-R) under the manufacturer’s instructions (AmpliTaq Gold, Invitrogen) with an annealing temperature of 55°C.

## Results

### Iturinic Lipopeptide Diversity at the Species Level

**FIGURE 2:**
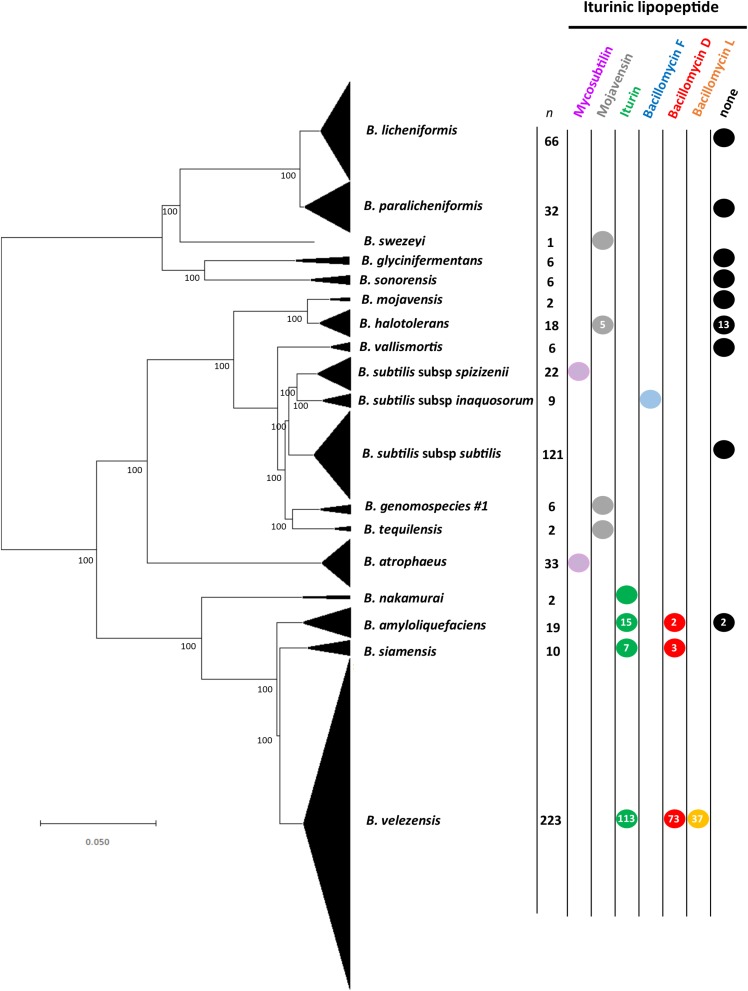
Neighbor-joining phylogeny showing iturinic lipopeptide occurrence across the *B. subtilis* species group. The tree was reconstructed from the core genomes of 584 strains. Bootstrap values >50%, based on 1,000 pseudoreplicates are indicated on branch points. The tree was rooted based on previous core genome phylogenies of the *B. subtilis* group (Dunlap et al., 2015b). Species in the “*Bacillus pumilus*” clade did not contain the cluster and were omitted from the figure to save space. The scale bar corresponds to 0.05 nucleotide substitutions per site.

Genome mining identified iturinic lipopeptide gene clusters in 11 species or subspecies from the *B. subtilis* species group (Figure 2). It was determined that one of the clades in the reconstructed species phylogeny contained an iturinic lipopeptide gene cluster, and this clade corresponds to a hitherto unknown species based on ANI calculations (<96% with known species; Richter and Rosselló-Móra, 2009). This clade will be referred to as “*Bacillus* genomospecies #1.” The results show that 8 of the 11 species in the *B. subtilis* species group only make one iturinic compound (Figure 2), although the analyses of the species that seemingly only make one compound are limited by the number of genomes available (i.e., *Bacillus tequilensis*, *Bacillus nakamurai*, *Bacillus swezeyi*, and “*Bacillus* genomospecies #1”). In the case of the remaining single compound producing strains (*Bacillus halotolerans*, *Bacillus subtilis* subsp. *spizizenii*, *Bacillus subtilis* subsp. *inaquosorum*, and *Bacillus atrophaeus*) we feel confident that they likely produce only one compound, as we analyzed at least 9 genomes per species (Supplementary Table S2). In nearly all instances, either all of the genomes examined for a given species had an iturinic lipopeptide gene cluster or all genomes of the species lacked the cluster entirely. The two exceptions were. *B. halotolerans* and *B. amyloliquefaciens*, for which 72% (13/18) of genomes and 11% (2/19) of genomes, respectively, lacked the cluster. Supplementary Table S2 shows the assignment of the iturinic lipopeptide for all genomes in which a gene cluster was present. No strain was found to contain more than one iturinic cluster. A protein BLAST search only identified three instances in which the cluster was found in a species other than a member of the *B. subtilis* species group; all of these were found in strains of *Paenibacillus larvae* (GenBank accession numbers: CP019655, CP020557, CP003355).

Two species were identified that make their own unique lipopeptide: *B. subtilis* subsp. *inaquosorum* is the only species to produce bacillomycin F, and *B. velezensis* is the only species known to produce bacillomycin L. In addition, *B. velezensis* produces the greatest variety of lipopeptides, as some strains produce iturin or bacillomycin D instead of bacillomycin L. It is interesting to note that all iturin producers fall within the same clade, which encompasses *B. amyloliquefaciens*, *B. nakamuari*, *B. siamensis*, and *B. velezensis.* This clade also contains the only producers of bacillomycin D and bacillomycin L. It was previously believed that *B. mojavensis* produced only mojavensin. However, the strains apparently belong to another closely related species, *B. halotolerans* (Dunlap et al., 2016a). In addition, all genomes that contained a cluster also contained the *sfp* gene required for NRPS activation, except for two strains; *B. atrophaeus* ATCC 9372-1 and 1013-2. These two strains are domesticated laboratory strains that have been reported to have large genome deletions from this portion of the genome (Gibbons et al., 2011).

### Iturinic Lipopeptide Diversity at the Strain Level

**FIGURE 3:**
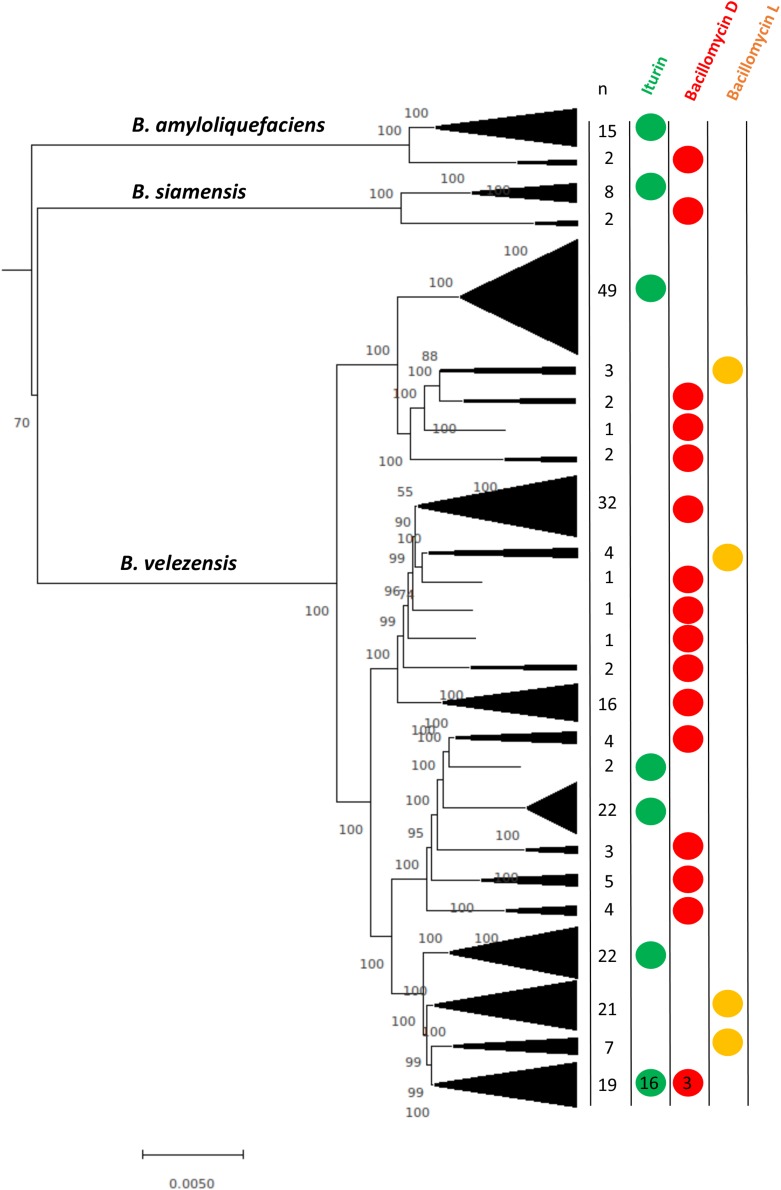
Neighbor-joining phylogeny showing iturinic lipopeptide occurrence among strains within the clade that encompasses *B. amyloliquefaciens*, *B. siamensis*, and *B. velezensis*. The tree was reconstructed from the core genomes of 250 strains. Bootstrap values >50%, based on 1,000 pseudoreplicates are indicated on branch points. The tree was rooted based on previous core genome phylogenies of the *B. subtilis* group (Dunlap et al., 2015b). The scale bar corresponds to 0.005 nucleotide substitutions per site.

Because *B. amyloliquefaciens*, *B. siamensis*, and *B. velezensis* each produce more than one lipopeptide, we determined the distribution of the lipopeptides within the species based on core genome phylogeny (Figure 3). The results show that the different lipopeptide gene clusters appear in the core-genome phylogeny in a clade-dependent manner, which suggests unique introductions of the biosynthetic clusters. For example, it appears that, on the basis of the core genome phylogeny, the bacillomycin L cluster was likely introduced into *B. velezensis* 3 or 4 times. In addition, the phylogeny in Figure 3 also indicates that *B. amyloliquefaciens* and *B. siamensis* encompass distinct strain lineages that possess the iturin and bacillomycin D clusters.

### Phylogeny Based on Conserved Cluster Gene, *ituA* and Synteny

**FIGURE 4:**
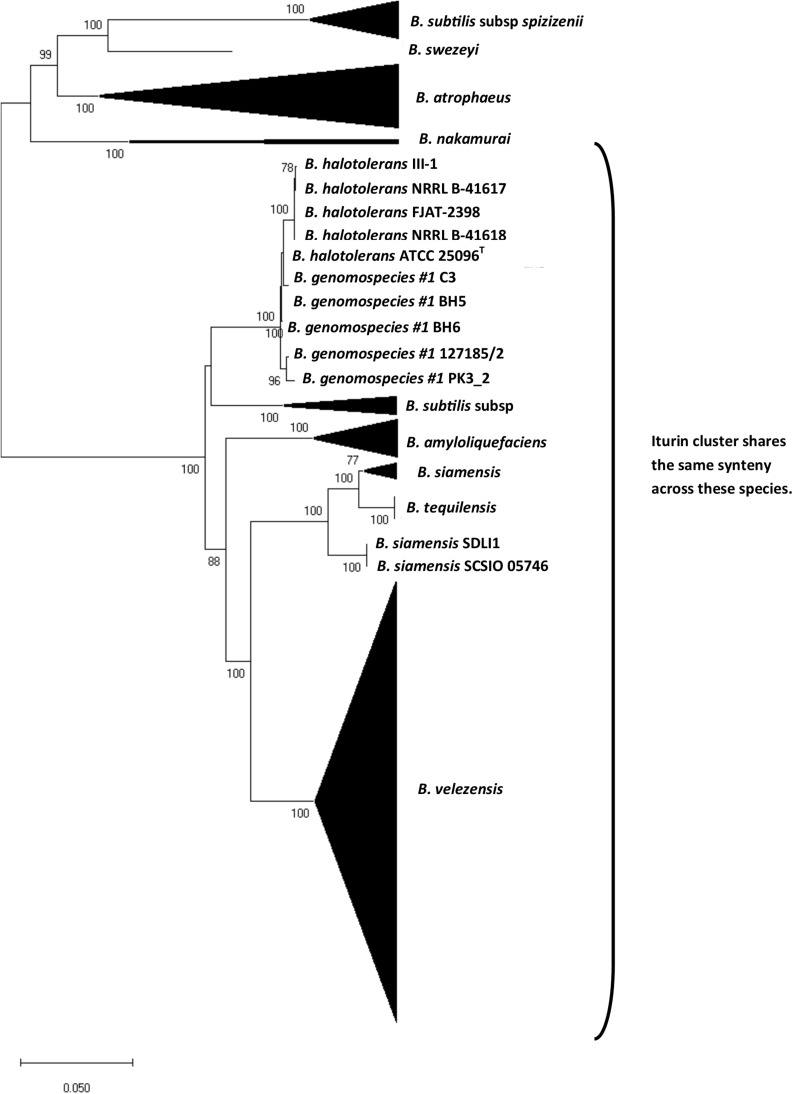
Neighbor-joining phylogenetic tree reconstructed from the partial *ituA* gene of iturinic lipopeptide producing strains from the *B. subtilis* group. Bootstrap values >50%, based on 1,000 pseudoreplicates are indicated on branch points. The scale bar corresponds to 0.05 nucleotide substitutions per site.

To better understand the evolutionary history of the iturin cluster in the *B. subtilis* species group, we conducted a phylogenetic analysis using a conserved region of the cluster corresponding to a segment of the *ituA* gene that is found in all genomes. The results show considerable incongruence between the *ituA* gene phylogeny (Figure 4) and core genome phylogeny (Figure 2). We also examined the syntenic relationships of the iturinic clusters across species and discovered that many are found in the same location in the genome (Figure 4). The few notable exceptions are the two mycosubtilin producers *B. subtilis* subsp. *spizizenii* and *B. atrophaeus.* The cluster in these two species also do not share synteny which suggests it likely came from a horizontal gene transfer event, rather than a shared ancestor. *B. swezeyi* also does not share synteny with any other species, which is not necessarily surprising because it is also the most distantly related strain possessing the cluster.

Given the incongruence between the partial *ituA* gene and the core genome phylogeny, we calculated the homology of the partial *ituA* gene and genomic ANI for representatives of each species. The results are provided in Table 1 and highlight several cases in which the partial *ituA* gene sequence and genomic ANI differ by more than 10% in comparisons involving the same species. For instance, the genomic ANI between *B. halotolerans* and *Bacillus* genomospecies #1 is 87.6%, whereas the partial *ituA* gene sequence identity is 99.1%. On the other hand, the opposite is also found in which the genomic ANI is much higher than the partial *ituA* gene sequence identity. For example, the genomic ANI is 94.5% whereas the partial *ituA* gene sequence identity is 81.9% between *B. subtilis* subsp. *spizizenii* and *B. subtilis* subsp. *inaquosorum*.

**TABLE 1:** Sequence similarity of the partial *ituA* gene between representative strains of iturinic lipopeptide producing species and genomic ANI values from comparisons of those same species.

**Species**	**1**	**2**	**3**	**4**	**5**	**6**	**7**	**8**	**9**	**10**	**11**	**12**
1*B. velezensis*		90.7	90.8	89.9	85.0	84.8	90.9	90.6	90.9	80.6	90.9	83.1
2 B. siamensis (iturin)	94.4		98.3	89.7	85.4	85.1	90.2	97.7	90.4	81.0	90.6	83.3
3 B. siamensis (bacD)	94.6	98.0		89.7	85.0	84.7	90.2	97.7	90.4	80.7	90.3	83.1
4 *B. amyloliquefaciens*	94.1	93.9	93.9		85.7	85.3	89.9	89.7	90.0	81.3	90.3	83.7
5*B. nakamurai*	86.5	86.0	86.2	86.6		92.2	86.3	84.9	86.3	85.8	87.0	89.7
6*B. atropheaus*	77.3	77.3	77.3	77.3	78.4		86.3	84.8	86.3	87.3	86.3	91.0
7*B. halotolerans*	77.0	77.1	77.1	77.2	77.7	80.6		90.5	99.1	82.0	93.5	84.4
8*B. tequilensis*	77.3	77.7	77.5	77.2	77.8	79.7	87.3		90.6	80.6	90.5	83.3
9*B. genomspecies 1*	77.1	77.4	77.1	77.1	77.6	79.8	87.6	93.6		81.8	93.5	83.7
10 *B. subtilis subspspizizenii*	77.1	77.0	77.3	77.1	77.5	80.0	87.9	92.6	93.6		81.9	87.9
11 *B. subtilis subspinaquosorum*	77.1	77.2	77.1	77.2	77.2	80.1	88.2	92.8	93.8	94.5		84.1
12 *B. swezeyi*	72.5	72.3	72.5	72.4	72.8	73.3	73.3	72.8	73.1	73.3	73.3	

*ituA sequence similarity less than genomic ANI-10%.*

*ituA sequence similarity greater than genomic ANI+10%.*

*The partial ituA gene data is in the upper right of the table and the genomic ANI data is in the lower left of the table.*

### Selective Primers to Determine Iturinic Cluster in *B. velezensis*

**FIGURE 5:**
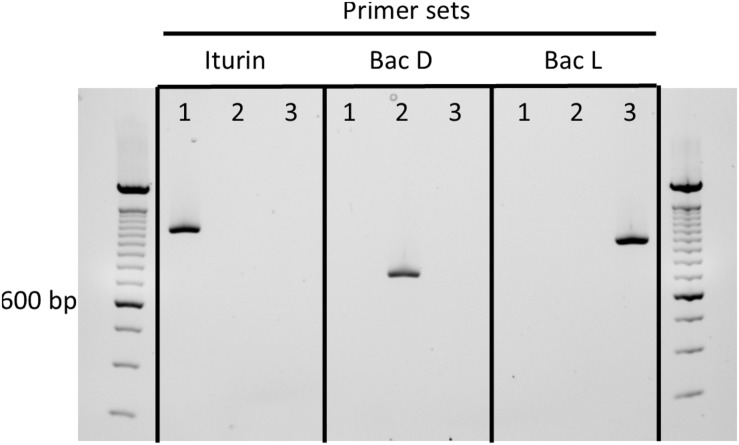
Agarose gel containing amplicons from selective primers sets to determine iturinic lipopeptide production in *B. velezensis* strains: (1) iturin, *B. velezensis* QST713, (2) bacillomycin D, *B. velezensis* FZB42, (3) bacillomycin L, *B. velezensis* KACC 18228. The iturin set is ituB-fwd and ituB-iturin-rev; the BacD set is ituB-fwd and ituB-bacD-rev; and the BacL set is ituB-fwd and ituB-R-bacL. A size ladder standard in 100 bp increments is included in the first and last unlabeled lanes, with a bold band corresponding to 600 bp.

Because *B. velezensis* strains are among the most well studied iturinic lipopeptide producers and the most commonly used for commercial purposes (Dunlap, 2019), we developed a rapid, diagnostic PCR assay that uses selective primers to amplify iturinic lipopeptide gene types that can be differentiated subsequently on the basis of amplicon size through agarose gel electrophoresis. The primer sets share the same forward primer, which targets the conserved third amino acid module (Figure 1). The reverse primers target the variable fourth amino acid module in the three different clusters (iturin A, bacillomycin D, and bacillomycin L). The primer sets yield a ∼1,113 bp band for iturin, ∼1,016 bp band for bacillomycin L and a ∼732 bp band for bacillomycin D. The end result is a simple diagnostic PCR assay that provides a rapid method to determine which specific lipopeptide compound is produced by a given strain (Figure 5).

### Mass Spectrometric Analysis of Iturinic Lipopeptides

Iturinic lipopeptides fragment through two dominant pathways (Supplementary Figure S1A), which differ based upon where the peptide ring breaks and creates a linear peptide. Stepwise fragmentation of the terminal ends of these linear peptides can then be used to assign amino acid constituents (Supplementary Figure S1A). When the opening of the cyclic lipopeptide occurs between the β-amino acid tail and the conserved Asn at AA#1, diagnostic ions are generated from the conserved AA#1-AA#2 fragments Asn-Tyr (*m*/*z* 278.11) and the fragment for Asn-Tyr-Asn (*m*/*z* 392.15); since these fragments do not contain the lipid tail, they are invariant across all six lipopeptides and alkyl chain variants (Supplementary Figures S1B–G). Additionally, an ion of 136.076 *m*/*z* is present in all iturinic lipopeptides and appears to be generated by a double fragmentation of the tyrosine substituent. The fragmentation of the parent ions also yields fragment ions that are dependent upon the chain length of the β-amino acid; a useful ion results from the loss of 42.01 Da from the β-amino acid providing the information required to determine the length of the chain (e.g., C14 fragments to 184.20; C15 fragments to 198.22, etc. Supplementary Figures S1B–G). These fragments facilitate the assignment of 14.02 Da changes to either the β-amino acid or to the cyclic peptides (i.e., Ser to Thr). When the opening of the cyclic peptide occurs at the proline residue, short peptide fragments can be used to assign the variable residues at AA#5-AA#6-AA#7 for Iturin A (Supplementary Figure S1B), mycosubtilin (Supplementary Figure S1C), bacillomycin F (Supplementary Figure S1F), or mojavensin A (Supplementary Figure S1G) where proline occupies the AA#5 position or AA-#4-AA#5-AA#6-AA#7 for bacillomycin D where the proline occupies the AA#4 position (Supplementary Figures S1A–E). Bacillomycin L lacks a proline; thus, it has a more uniform fragmentation pattern across the β-amino acid (Supplementary Figure S1D).

### Comparison to NRPS Substrate Predictor Software

Proteins representing the *ituA*, *ituB*, and *ituC* genes from the 15 cluster groups were subjected to substrate prediction. The software successfully predicted substrate specificity of amino acid modules in 101 out 105 modules (Supplementary Table S3). The only missed predictions were for Bacillomycin D and Bacillomycin L *ituB* genes, where the observed glutamate was predicted to be glutamine.

## Discussion

It is well known that iturinic lipopeptides possess strong antifungal activity (Mhammedi et al., 1982; Peypoux et al., 1986; Ongena and Jacques, 2008; Ma et al., 2012; Guardado-Valdivia et al., 2018) and induce defense responses in plants (Farace et al., 2015; Park et al., 2016; Wu et al., 2018) which, in turn, has led to the commercialization of many *Bacillus* strains that manufacture these compounds (Dunlap, 2019). Interestingly, no known resistance mechanisms to iturinic lipopeptides have been reported in fungi. It is not clear if changes in the gene cluster encoding iturinic lipopeptides are the result of interactions with other fungi or plants, or due to an intrinsic factor such as differential genome dynamics (Rooney and Ward, 2005). Based on gene synteny, it can be inferred that the cluster was either introduced into, or evolved *de novo* in, the *B. subtilis* species group after *Bacillus licheniformis* and *Bacillus pumilus* clades arose (Figure 2). However, the sporadic distribution among the remaining members further suggests that either more than one introduction occurred or that the gene cluster evolves under a birth-and-death process (Nei and Rooney, 2005), although additional studies are needed to confirm the evolutionary processes. The fact that two mycosubtilin producers, *B. atrophaeus* and *B. subtilis* subsp. *spizizenii*, possess the cluster but do not share synteny with other members in the clade or with each other is consistent with the multiple introduction hypothesis. This is further supported by the incongruence of the partial *ituA* gene phylogeny (Figure 4) and the core genome phylogeny (Figure 2). On the other hand, the only other species that has been identified to possess an iturinic lipopeptide gene cluster is *P. larvae* (Sood et al., 2014), in which the gene cluster is in three genomes. The product of these *P. larvae* clusters (paenilarvin) shares the same cyclic heptapeptide sequence as mojavensin and only differs in the alkyl chain length (Sood et al., 2014). Although it is currently not possible to conclude with certainty the direction in which the horizontal transfer event occurred based on available data (i.e., from *B. subtilis* species group into *P. larvae*, or vice-versa), the fact that the gene cluster is found among 330 genomes and conserved across multiple species in the *B. subtilis* species group in contrast to being found in only three genomes in only one species of *Paenibacillus* tilts the evidence in favor of the transfer occurring from a *Bacillus* species into *Paenibacillus*, as the amount breadth of diversity seen in the former would have taken a substantial amount of time to have been generated. That being the case, it further suggests that the gene cluster arose in *Bacillus* (most likely only once) and subsequently diversified according to a birth-and-death process in light of its sporadic phylogenetic distribution across the *B. subtilis* species group (Figure 2). Birth-and-death evolution is one possible explanation how a compound such as mojavensin (Ma et al., 2012) can be made by various species that are not sister taxa, and it also explains why some species possess an iturinic lipopeptide gene cluster whereas others do not. This study is also agreement with a recent study that looked at the presence or absence of iturinic cluster without chemotype assignment for five of these species (Torres Manno et al., 2019). One exception between the studies we noted is Torres Manno et al. (2019) reports one *Bacillus velezensis* strain (TF28) that does not contain an iturinic cluster, while our study excluded the strain because it contained too many frameshifted gene, including ituB. The genome for strain TF28 appears to suffer from some assembly issues with frameshifted genes in both the iturinic cluster and in the cluster that produces the antifungal, fengycin. The strain has been reported to make both iturin (Zhang et al., 2012) and fengycin (Liu et al., 2018).

Several studies have recently reported antifungal lipopeptides in *B. atrophaeus*, but none have identified the active compound (Zhang et al., 2013, 2016; Chen et al., 2017; Guardado-Valdivia et al., 2018). This study was first to identify *B. atrophaeus* as a producer of mycosubtilin. Furthermore, it is the first to report that *B. swezeyi* (Dunlap et al., 2017) and *B. nakamurai* (Dunlap et al., 2016c) are capable of producing iturinic lipopeptides that correspond to mojavensin and iturin, respectively. In addition, this study identified that *B. subtilis* subsp. *spizizenii* and *B. subtilis* subsp *inaquosorum* only make one lipopeptide each, mycosubtilin and bacillomycin F, respectively. Recent studies based on whole genome comparisons and ANI suggest that *B. subtilis* subsp. *spizizenii* and *B. subtilis* subsp. *inaquosorum* should be promoted to species status based on established ANI guidelines of species delineation (Yi et al., 2014; Brito et al., 2018; Knight et al., 2018). The primary reason these strains were relegated to subspecies status was a lack of distinguishing phenotypes, as well as a lack of complete genome information and guidelines on how to use it for taxonomy (Rooney et al., 2009). The findings of this study support their promotion to species-level status.

Although several iturinic peptides can be putatively identified by their molecular weight (bacillomycin L, bacillomycin D, and mojavensin A), others cannot due to the isomeric nature of the amino acid substitutions and variation of alkyl tail lengths of the lipid portion of the molecule. Despite the potential for differences between molecular masses, assignments can be confounded by the presence of molecules with masses close to the iturinic peptides. This effect is pronounced when low-resolution mass analyzers are used in the absence of chromatographic separations. Therefore, liquid chromatography coupled to tandem mass spectrometry is a powerful tool to confirm the presence and identity of iturinic lipopeptides. The use of quadropole-time-of-flight or HCD cells with orbitrap mass spectrometers are particularly useful for tandem mass spectrometry due to their high mass accuracy and the ability to collect fragment data from the lower mass ranges to identify the characteristics of the amino acid substitutions. In this study, the use of HCD with orbitrap detection led to confirmation of the assignments of all iturinic peptide types. The comprehensive fragmentation reference table provided (Supplementary Figure S1A) should allow easy assignment of these compounds in future studies. In addition, this study should provide additional reference material to enhance the accuracy of NRPS substrate prediction software.

Lastly, we developed selective primers to identify the iturinic lipopeptides produced by *B. velezensis*. Part of the motivation for the development of these primers was the misuse or over interpretation of other commonly reported primers. Some of the most commonly used primers to detect iturin producers target the part of the cluster conserved among all iturinic lipopeptides, such as *ituA* (Tsuge et al., 2005) or *ituD* (Hsieh et al., 2008), so they are not selective for iturin specifically. Primers that are commonly used to determine bacillomycin D producers target the *ituC* gene (Ramarathnam et al., 2007), which shares high homology with bacillomycin L. This often leads to incorrect reporting of compound production capabilities of certain strains. Therefore, it is important to note that the primers developed in this study have only been validated for *B. velezensis* strains; their application to other *Bacillus* species must be validated before they can be reliably applied.

## Data Availability

Publicly available datasets were analyzed in this study. This data can be found here: Refseq Bacillus genomes on GenBank (see Supplementary Material).

## Author Contributions

CD conceived the study and conducted the bioinformatics analysis. MB conducted the mass spectroscopy experimental work and analysis. AR interpreted the evolutionary relationships. All authors wrote, read, and reviewed the final draft of the manuscript.

## Conflict of Interest Statement

The authors declare that the research was conducted in the absence of any commercial or financial relationships that could be construed as a potential conflict of interest.
