# Supplementary material for: Iturinic Lipopeptide Diversity in the Bacillus subtilis Species Group – Important Antifungals for Plant Disease Biocontrol Applications
Source: Front Microbiol. 2019 Aug 7;10:1794. doi: 10.3389/fmicb.2019.01794 (PMC6693446; doi:10.3389/fmicb.2019.01794)

SUPPLEMENTAL MATERIALS

for

Iturinic lipopeptide diversity in the *Bacillus subtilis* species group – important antifungals for plant disease biocontrol applications

Christopher A. Dunlap[a]*, Michael J.Bowman[b] and Alejandro P. Rooney[a]

[a]Crop Bioprotection and [b]Bioenergy Research Units, National Center for Agricultural Utilization Research, Agricultural Research Service, United States Department of Agriculture, 1815 North University Street, Peoria, Illinois 61604

*Corresponding author: Christopher.dunlap@ars.usda.gov

Table S1. Genes to BLAST to determine iturinic lipopeptide by species

Bacillus amyloliquefaciens

ItuB iturin (DSM 7)

https://www.ncbi.nlm.nih.gov/nuccore/FN597644.1?report=fasta&from=1976456&to=1992541

ItuC iturin (DSM 7)

https://www.ncbi.nlm.nih.gov/nuccore/FN597644.1?report=fasta&from=1968515&to=1976365

ItuB bacillomycin D (CHCC26933)

https://www.ncbi.nlm.nih.gov/nuccore/PQWK01000002.1?report=fasta&from=745894&to=761985

ItuC bacillomycin D (CHCC26933)

https://www.ncbi.nlm.nih.gov/nuccore/PQWK01000002.1?report=fasta&from=737948&to=745810

Bacillus atrophaeus

ItuB mycosubtilin

https://www.ncbi.nlm.nih.gov/nuccore/NZ_CP021500.1?report=fasta&from=2107540&to=2123631

ItuC mycosubtilin

https://www.ncbi.nlm.nih.gov/nuccore/NZ_CP021500.1?report=fasta&from=2099652&to=2107460

Bacillus genomospecies #1

ItuB mojavensin

https://www.ncbi.nlm.nih.gov/nuccore/MIZD01000002.1?report=fasta&from=430578&to=446663

ItuC mojavensin

https://www.ncbi.nlm.nih.gov/nuccore/MIZD01000002.1?report=fasta&from=422673&to=430487\

Bacillus halotolerans

ItuB mojavensin

https://www.ncbi.nlm.nih.gov/nuccore/NZ_MBQV01000011.1?report=fasta&from=1029620&to=1045702

ItuC mojavensin

https://www.ncbi.nlm.nih.gov/nuccore/NZ_MBQV01000011.1?report=fasta&from=1021715&to=1029529

Bacillus nakamurai

ItuB iturin

https://www.ncbi.nlm.nih.gov/nuccore/LSAZ01000041.1?report=fasta&from=465938&to=482026

ItuC iturin

https://www.ncbi.nlm.nih.gov/nuccore/LSAZ01000041.1?report=fasta&from=458007&to=465854

Bacillus siamensis

ItuB iturin (7551)

https://www.ncbi.nlm.nih.gov/nuccore/NPCI01000001.1?report=fasta&from=43522&to=59607

ItuC iturin (7551)

https://www.ncbi.nlm.nih.gov/nuccore/NPCI01000001.1?report=fasta&from=59696&to=67552

ItuB bacillomycin D (scd15)

https://www.ncbi.nlm.nih.gov/nuccore/PEKS01000277.1?report=fasta&from=19437&to=35525

ItuC bacillomycin D (scd15)

https://www.ncbi.nlm.nih.gov/nuccore/PEKS01000277.1?report=fasta&from=11491&to=19353

Bacillus subtilis* subsp *inaquosorum

ItuB bacillomycin F

https://www.ncbi.nlm.nih.gov/nuccore/CP029465.1?report=fasta&from=2095894&to=2111987

ItuC bacillomycin F

https://www.ncbi.nlm.nih.gov/nuccore/CP029465.1?report=fasta&from=2087978&to=2095804

Bacillus subtilis* subsp *spizizenii

ItuB mycosubtilin

https://www.ncbi.nlm.nih.gov/nuccore/CP002183.1?report=fasta&from=1919861&to=1935958

ItuC mycosubtilin

https://www.ncbi.nlm.nih.gov/nuccore/CP002183.1?report=fasta&from=1911931&to=1919763

Bacillus swezeyi

ItuB mojavensin

https://www.ncbi.nlm.nih.gov/nuccore/MTJL01000042.1?report=fasta&from=50947&to=67047

ItuC mojavensin

https://www.ncbi.nlm.nih.gov/nuccore/MTJL01000042.1?report=fasta&from=67145&to=74956

Bacillus tequilensis

ItuB mojavensin

https://www.ncbi.nlm.nih.gov/nuccore/UAQB01000027.1?report=fasta&from=181959&to=198053

ItuC mojavensin

https://www.ncbi.nlm.nih.gov/nuccore/UAQB01000027.1?report=fasta&from=174053&to=181870

Bacillus velezensis

ItuB bacillomycin L (KACC 18228)

https://www.ncbi.nlm.nih.gov/nuccore/LLZA01000001.1?report=fasta&from=44524&to=60651

ItuC bacillomycin L (KACC 18228)

https://www.ncbi.nlm.nih.gov/nuccore/LLZA01000001.1?report=fasta&from=60735&to=68594

ItuB bacillomycin D (FZB42)

https://www.ncbi.nlm.nih.gov/nuccore/CP000560.1?report=fasta&from=1879116&to=1895207

ItuC bacillomycin D (FZB42)

https://www.ncbi.nlm.nih.gov/nuccore/CP000560.1?report=fasta&from=1871172&to=1879031

ItuB iturin (AS43.3)

https://www.ncbi.nlm.nih.gov/nuccore/CP003838.1?report=fasta&from=1940687&to=1956775

ItuC iturin (AS43.3)

https://www.ncbi.nlm.nih.gov/nuccore/CP003838.1?report=fasta&from=1932742&to=1940421

Table S2. Iturinic cluster assignment for all genomes analyzed.

Species	Isolate	Assignment	confirmed with MS/MS
Bacillus amyloliquefaciens	ATCC 13952	Iturin	X
Bacillus amyloliquefaciens	B425	Iturin	
Bacillus amyloliquefaciens	CHCC26933	Bacillomycin D	
Bacillus amyloliquefaciens	CMW1	none	
Bacillus amyloliquefaciens	DSM 7	Iturin	(11)
Bacillus amyloliquefaciens	HK1	Iturin	
Bacillus amyloliquefaciens	K2	Iturin	
Bacillus amyloliquefaciens	KCP2	Iturin	
Bacillus amyloliquefaciens	LL3	Iturin	(12)
Bacillus amyloliquefaciens	LMG 2099	Iturin	
Bacillus amyloliquefaciens	MT45	Iturin	
Bacillus amyloliquefaciens	NRRL 942	Iturin	
Bacillus amyloliquefaciens	RD7-7	Bacillomycin D	
Bacillus amyloliquefaciens	SRCM101266	Iturin	
Bacillus amyloliquefaciens	SRCM101267	Iturin	
Bacillus amyloliquefaciens	SRCM101294	none	
Bacillus amyloliquefaciens	TA208	Iturin	
Bacillus amyloliquefaciens	XH7	Iturin	
Bacillus amyloliquefaciens	YP6	Iturin	
Bacillus atrophaeus	1942	Mycosubtilin	
Bacillus atrophaeus	930029	Mycosubtilin	
Bacillus atrophaeus	1013-1	Mycosubtilin	
Bacillus atrophaeus	1013-2	Mycosubtilin	
Bacillus atrophaeus	ATCC 49822-1	Mycosubtilin	
Bacillus atrophaeus	ATCC 49822-2	Mycosubtilin	
Bacillus atrophaeus	ATCC 9372-1	Mycosubtilin	
Bacillus atrophaeus	ATCC 9372-2	Mycosubtilin	
Bacillus atrophaeus	B4144	Mycosubtilin	
Bacillus atrophaeus	B4144_201601	Mycosubtilin	
Bacillus atrophaeus	BA59	Mycosubtilin	
Bacillus atrophaeus	BACI051-E	Mycosubtilin	
Bacillus atrophaeus	BACI051-N	Mycosubtilin	
Bacillus atrophaeus	BSS	Mycosubtilin	
Bacillus atrophaeus	C89	Mycosubtilin	
Bacillus atrophaeus	CKL1	Mycosubtilin	
Bacillus atrophaeus	Detrick-1	Mycosubtilin	
Bacillus atrophaeus	Detrick-2	Mycosubtilin	
Bacillus atrophaeus	Detrick-3	Mycosubtilin	
Bacillus atrophaeus	DJHJ8	Mycosubtilin	
Bacillus atrophaeus	Dugway	Mycosubtilin	
Bacillus atrophaeus	GBSC56	Mycosubtilin	
Bacillus atrophaeus	GQJK17	Mycosubtilin	

Table S2. Iturinic cluster assignment for all genomes analyzed.

Species	Isolate	Assignment	confirmed with MS/MS
Bacillus atrophaeus	LSSC3	Mycosubtilin	
Bacillus atrophaeus	NBRC 15539	Mycosubtilin	
Bacillus atrophaeus	NMTD54	Mycosubtilin	
Bacillus atrophaeus	NRRL NRS 213	Mycosubtilin	X
Bacillus atrophaeus	NRRL NRS-1221	Mycosubtilin	X
Bacillus atrophaeus	NRRL NRS-650	Mycosubtilin	X
Bacillus atrophaeus	NRS 1221	Mycosubtilin	X
Bacillus atrophaeus	SCRM101359	Mycosubtilin	
Bacillus atrophaeus	TS1	Mycosubtilin	
Bacillus atrophaeus	UCMB-5137	Mycosubtilin	
Bacillus genomospecies #1	127185/2	Mojavensin	
Bacillus genomospecies #1	BH5	Mojavensin	
Bacillus genomospecies #1	BH6	Mojavensin	
Bacillus genomospecies #1	C3	Mojavensin	
Bacillus genomospecies #1	ND23	Mojavensin	
Bacillus genomospecies #1	PK3_2	Mojavensin	
Bacillus halotolerans	36	none	
Bacillus halotolerans	72	none	
Bacillus halotolerans	86	none	
Bacillus halotolerans	ATCC 25096	Mojavensin	X
Bacillus halotolerans	B19	none	
Bacillus halotolerans	DGL6	none	
Bacillus halotolerans	FJAT-2398	Mojavensin	
Bacillus halotolerans	III-1	Mojavensin	
Bacillus halotolerans	LNXM37	none	
Bacillus halotolerans	LNXM78	none	
Bacillus halotolerans	MS50-18A	none	
Bacillus halotolerans	NRRL B-41617	Mojavensin	X
Bacillus halotolerans	NRRL B-41618	Mojavensin	X
Bacillus halotolerans	RRC 101	none	
Bacillus halotolerans	RSCu-8D	none	
Bacillus halotolerans	V44_23b	none	
Bacillus halotolerans	V48-19	none	
Bacillus halotolerans	ZB201702	none	
Bacillus nakamurai	NRRL B-41091	Iturin	X
Bacillus nakamurai	NRRL B-41092	Iturin	X
Bacillus siamensis	7551	Iturin	
Bacillus siamensis	JFL15	Iturin	(15)
Bacillus siamensis	JJC33M	Iturin	

Table S2. Iturinic cluster assignment for all genomes analyzed.

Species	Isolate	Assignment	confirmed with MS/MS
Bacillus siamensis	KACC 16244	Iturin	
Bacillus siamensis	KCTC 13613	Iturin	X
Bacillus siamensis	SCSIO 05746	Bacillomycin D	(5)
Bacillus siamensis	sdc15	Bacillomycin D	
Bacillus siamensis	SDLI1	Bacillomycin D	
Bacillus siamensis	SRCM100169	Iturin	
Bacillus siamensis	XY18	Iturin	X
Bacillus subtilis subsp inaquosorum	ATCC 55406	Bacillomycin F	X
Bacillus subtilis subsp inaquosorum	CU1	Bacillomycin F	
Bacillus subtilis subsp inaquosorum	CW14	Bacillomycin F	
Bacillus subtilis subsp inaquosorum	DE111	Bacillomycin F	(14)
Bacillus subtilis subsp inaquosorum	HUBIOL-II	Bacillomycin F	(14)
Bacillus subtilis subsp inaquosorum	KCTC 13429	Bacillomycin F	X
Bacillus subtilis subsp inaquosorum	KR2-7	Bacillomycin F	
Bacillus subtilis subsp inaquosorum	SB-14	Bacillomycin F	
Bacillus subtilis subsp inaquosorum	SN1	Bacillomycin F	
Bacillus subtilis subsp *spizizenii*	1731	Mycosubtilin	
Bacillus subtilis subsp *spizizenii*	AS2	Mycosubtilin	
Bacillus subtilis subsp *spizizenii*	ATCC 6633	Mycosubtilin	(13)
Bacillus subtilis subsp *spizizenii*	BST	Mycosubtilin	
Bacillus subtilis subsp *spizizenii*	DSM 1297	Mycosubtilin	
Bacillus subtilis subsp *spizizenii*	GM2	Mycosubtilin	
Bacillus subtilis subsp *spizizenii*	HUK15	Mycosubtilin	
Bacillus subtilis subsp *spizizenii*	Ia1a	Mycosubtilin	
Bacillus subtilis subsp *spizizenii*	JCM 2499	Mycosubtilin	
Bacillus subtilis subsp *spizizenii*	JRS7	Mycosubtilin	
Bacillus subtilis subsp *spizizenii*	MJ01	Mycosubtilin	
Bacillus subtilis subsp *spizizenii*	NCTC 10400	Mycosubtilin	
Bacillus subtilis subsp *spizizenii*	NRRL B-23049	Mycosubtilin	X
Bacillus subtilis subsp *spizizenii*	NRRL B-51215	Mycosubtilin	X
Bacillus subtilis subsp *spizizenii*	NRRL NRS 231	Mycosubtilin	X
Bacillus subtilis subsp *spizizenii*	RFWG1A3	Mycosubtilin	
Bacillus subtilis subsp *spizizenii*	RFWG1A4	Mycosubtilin	
Bacillus subtilis subsp *spizizenii*	RFWG4C10	Mycosubtilin	
Bacillus subtilis subsp *spizizenii*	RFWG5B15	Mycosubtilin	
Bacillus subtilis subsp *spizizenii*	T30	Mycosubtilin	
Bacillus subtilis subsp *spizizenii*	TU-B-10	Mycosubtilin	
Bacillus subtilis subsp *spizizenii*	W23	Mycosubtilin	
Bacillus swezeyi	NRRL B-41294	Mojavensin	X

Table S2. Iturinic cluster assignment for all genomes analyzed.

Species	Isolate	Assignment	confirmed with MS/MS
Bacillus tequilensis	KCTC 13622	Mojavensin	X
Bacillus tequilensis	NCTC 13306	Mojavensin	X
Bacillus velezensis	83	Bacillomycin D	(6)
Bacillus velezensis	157	Bacillomycin D	
Bacillus velezensis	275	Iturin	
Bacillus velezensis	916	Bacillomycin L	(4)
Bacillus velezensis	7899	Iturin	
Bacillus velezensis	10075	Bacillomycin D	
Bacillus velezensis	11B91	Bacillomycin L	
Bacillus velezensis	131-4	Iturin	
Bacillus velezensis	1B-23	Iturin	
Bacillus velezensis	2A-2B	Bacillomycin L	
Bacillus velezensis	3A-25B	Bacillomycin D	
Bacillus velezensis	5B6	Bacillomycin D	
Bacillus velezensis	5RB	Bacillomycin L	
Bacillus velezensis	7537-G2	Bacillomycin D	
Bacillus velezensis	7551-1	Iturin	
Bacillus velezensis	7551-2	Iturin	
Bacillus velezensis	7586-G	Iturin	
Bacillus velezensis	8-2	Iturin	
Bacillus velezensis	9912D	Bacillomycin D	
Bacillus velezensis	9D-6	Iturin	
Bacillus velezensis	A6	Bacillomycin D	
Bacillus velezensis	AGVL-005	Iturin	
Bacillus velezensis	AH159-1	Iturin	
Bacillus velezensis	ALB65	Bacillomycin D	
Bacillus velezensis	ALB69	Bacillomycin D	
Bacillus velezensis	ANSB01E	Iturin	
Bacillus velezensis	AP183	Iturin	
Bacillus velezensis	AP194	Iturin	
Bacillus velezensis	AP214	Iturin	
Bacillus velezensis	AS43.3	Iturin	(10)
Bacillus velezensis	ATCC 12321	Bacillomycin D	
Bacillus velezensis	ATCC 19217	Bacillomycin D	
Bacillus velezensis	B-1	Iturin	
Bacillus velezensis	B15	Iturin	
Bacillus velezensis	B1895	Iturin	
Bacillus velezensis	B25	Iturin	
Bacillus velezensis	B26	Bacillomycin L	
Bacillus velezensis	B4140	Bacillomycin L	
Bacillus velezensis	B5	Iturin	
Bacillus velezensis	B6	Bacillomycin L	

Table S2. Iturinic cluster assignment for all genomes analyzed.

Species	Isolate	Assignment	confirmed with MS/MS
Bacillus velezensis	B946	Iturin	
Bacillus velezensis	B9601-Y2	Bacillomycin D	
Bacillus velezensis	Bac57	Bacillomycin L	
Bacillus velezensis	BE2	Iturin	
Bacillus velezensis	BH072	Bacillomycin D	
Bacillus velezensis	BIM B-439D	Bacillomycin L	
Bacillus velezensis	Bs006	Iturin	
Bacillus velezensis	BS-37	Bacillomycin L	
Bacillus velezensis	Bs-916	Bacillomycin L	(2)
Bacillus velezensis	BTLK6A (K6A)	Iturin	
Bacillus velezensis	BTS 4 (R3)	Iturin	
Bacillus velezensis	BUU 004	Iturin	
Bacillus velezensis	C2	Bacillomycin D	
Bacillus velezensis	CBMB205	Iturin	
Bacillus velezensis	CBMC205	Iturin	
Bacillus velezensis	CC09	Iturin	
Bacillus velezensis	CC178	Bacillomycin D	
Bacillus velezensis	CE2	Iturin	
Bacillus velezensis	CFSAN034338	Iturin	
Bacillus velezensis	CFSAN034339	Iturin	
Bacillus velezensis	CFSAN034340	Iturin	
Bacillus velezensis	CGMCC 11640	Iturin	
Bacillus velezensis	CH13	Bacillomycin D	
Bacillus velezensis	CHCC26801	Bacillomycin D	
Bacillus velezensis	CMT-6	Iturin	
Bacillus velezensis	CN026	Bacillomycin D	
Bacillus velezensis	Co1-6	Iturin	
Bacillus velezensis	CS1.10S	Bacillomycin L	
Bacillus velezensis	D2-2	Bacillomycin L	
Bacillus velezensis	DC-12	Bacillomycin D	
Bacillus velezensis	DJFZ40	Bacillomycin D	
Bacillus velezensis	DKU_NT_04	Bacillomycin D	
Bacillus velezensis	DR-08	Iturin	
Bacillus velezensis	DSYZ	Iturin	
Bacillus velezensis	EBL11	Bacillomycin L	
Bacillus velezensis	EGD-AQ14	Bacillomycin L	
Bacillus velezensis	F11	Iturin	
Bacillus velezensis	FH17	Bacillomycin D	
Bacillus velezensis	Fito_F321	Iturin	
Bacillus velezensis	FKM10	Iturin	
Bacillus velezensis	FS001	Iturin	
Bacillus velezensis	FTC01	Iturin	
Bacillus velezensis	FZB42	Bacillomycin D	(7)

Table S2. Iturinic cluster assignment for all genomes analyzed.

Species	Isolate	Assignment	confirmed with MS/MS
Bacillus velezensis	G341	Bacillomycin L	(1)
Bacillus velezensis	GB03	Bacillomycin D	
Bacillus velezensis	GB1	Bacillomycin D	
Bacillus velezensis	GBSW11	Bacillomycin L	
Bacillus velezensis	GD4a	Bacillomycin D	
Bacillus velezensis	GF610	Iturin	
Bacillus velezensis	GFP-2	Bacillomycin D	
Bacillus velezensis	GH1-13	Bacillomycin D	
Bacillus velezensis	GQJK49	Iturin	
Bacillus velezensis	GR4-5	Bacillomycin D	
Bacillus velezensis	GYL4	Bacillomycin D	
Bacillus velezensis	GZB	Bacillomycin D	
Bacillus velezensis	H57	Bacillomycin L	
Bacillus velezensis	HB-26	Iturin	
Bacillus velezensis	HJ18-4	Iturin	
Bacillus velezensis	Hx05	Iturin	
Bacillus velezensis	IT-45	Iturin	
Bacillus velezensis	J01	Iturin	
Bacillus velezensis	J-5	Iturin	
Bacillus velezensis	J7-1	Iturin	
Bacillus velezensis	JJ-D34	Iturin	
Bacillus velezensis	JK	Iturin	
Bacillus velezensis	JRS5	Bacillomycin D	
Bacillus velezensis	JS25R	Bacillomycin D	
Bacillus velezensis	JT3-1	Iturin	
Bacillus velezensis	JTYP2	Iturin	
Bacillus velezensis	JW	Bacillomycin L	
Bacillus velezensis	Jxnu-18	Bacillomycin L	
Bacillus velezensis	Jxnuwx-1	Iturin	
Bacillus velezensis	K26	Bacillomycin D	
Bacillus velezensis	KACC 13105	Iturin	
Bacillus velezensis	KACC 18228	Bacillomycin L	X
Bacillus velezensis	KD1	Bacillomycin L	
Bacillus velezensis	KHG19	Bacillomycin L	
Bacillus velezensis	L-1	Iturin	
Bacillus velezensis	LABIM40	Bacillomycin L	
Bacillus velezensis	LB002	Iturin	
Bacillus velezensis	LDO2	Iturin	
Bacillus velezensis	LFB112	Iturin	
Bacillus velezensis	L-H15	Iturin	
Bacillus velezensis	LK7	Iturin	
Bacillus velezensis	LM2303	Iturin	
Bacillus velezensis	LPL-K103	Bacillomycin L	

Table S2. Iturinic cluster assignment for all genomes analyzed.

Species	Isolate	Assignment	confirmed with MS/MS
Bacillus velezensis	L-S60	Iturin	
Bacillus velezensis	LS69	Iturin	
Bacillus velezensis	Lzh-a42	Bacillomycin D	
Bacillus velezensis	M27	Iturin	
Bacillus velezensis	M49	Iturin	
Bacillus velezensis	M75	Iturin	
Bacillus velezensis	MBE1283	Iturin	
Bacillus velezensis	MG33	Iturin	
Bacillus velezensis	MG43	Bacillomycin L	
Bacillus velezensis	MH-2	Bacillomycin D	
Bacillus velezensis	MH25	Iturin	
Bacillus velezensis	MRC 16791	Bacillomycin D	
Bacillus velezensis	MRC 5958	Bacillomycin D	
Bacillus velezensis	NAU-B3	Bacillomycin D	
Bacillus velezensis	NB91	Iturin	
Bacillus velezensis	NBIF-001	Iturin	
Bacillus velezensis	NBIF-004	Iturin	
Bacillus velezensis	NJAU-Z9	Iturin	
Bacillus velezensis	NJN6	Iturin	(8)
Bacillus velezensis	NKG-1	Iturin	
Bacillus velezensis	NKYL29	Bacillomycin D	
Bacillus velezensis	NRRL B-23189	Iturin	
Bacillus velezensis	NRRL B-23247	Iturin	
Bacillus velezensis	NRRL B-41580	Bacillomycin D	X
Bacillus velezensis	NRRL B-4257	Bacillomycin D	
Bacillus velezensis	NRRL B-51623	Bacillomycin L	X
Bacillus velezensis	NRRL NRS-173	Iturin	
Bacillus velezensis	NWUMFk_BS10.5	Bacillomycin D	
Bacillus velezensis	NY12-2	Bacillomycin D	
Bacillus velezensis	OB9	Bacillomycin L	
Bacillus velezensis	OEE1	Iturin	
Bacillus velezensis	OSY-GA1	Iturin	
Bacillus velezensis	OSY-S3	Bacillomycin L	
Bacillus velezensis	P42	Bacillomycin L	
Bacillus velezensis	P7	Bacillomycin D	
Bacillus velezensis	Pc3	Iturin	
Bacillus velezensis	PEBA20	Bacillomycin D	
Bacillus velezensis	PG12	Iturin	
Bacillus velezensis	QST713	Iturin	X
Bacillus velezensis	RC 218	Iturin	(9)
Bacillus velezensis	RHNK22	Iturin	
Bacillus velezensis	RUPDJ	Iturin	
Bacillus velezensis	S141	Bacillomycin L	

Table S2. Iturinic cluster assignment for all genomes analyzed.

Species	Isolate	Assignment	confirmed with MS/MS
Bacillus velezensis	S3-1	Iturin	
Bacillus velezensis	S499	Iturin	
Bacillus velezensis	SB1216	Bacillomycin L	
Bacillus velezensis	SB-9	Iturin	
Bacillus velezensis	SCDB 291	Bacillomycin D	
Bacillus velezensis	SCGB 1	Bacillomycin D	
Bacillus velezensis	SCGB 574	Bacillomycin D	
Bacillus velezensis	SGAir0473	Bacillomycin D	
Bacillus velezensis	SK007	Iturin	
Bacillus velezensis	SK19.001	Bacillomycin D	
Bacillus velezensis	SPZ1	Bacillomycin D	
Bacillus velezensis	SQR9	Bacillomycin D	
Bacillus velezensis	SRCM100072	Bacillomycin D	
Bacillus velezensis	SRCM100730	Bacillomycin D	
Bacillus velezensis	SRCM100731	Bacillomycin D	
Bacillus velezensis	SRCM101413	Bacillomycin D	
Bacillus velezensis	SRCM103616	Bacillomycin D	
Bacillus velezensis	SRCM103639	Bacillomycin D	
Bacillus velezensis	SRCM103691	Bacillomycin D	
Bacillus velezensis	SRCM103788	Bacillomycin D	
Bacillus velezensis	SSBW-10	Bacillomycin D	
Bacillus velezensis	SSBW-18	Bacillomycin D	
Bacillus velezensis	SSBW-19	Bacillomycin D	
Bacillus velezensis	SSBW-2	Bacillomycin D	
Bacillus velezensis	SSBW-8	Bacillomycin D	
Bacillus velezensis	sx01604	Iturin	
Bacillus velezensis	SYBC H47	Bacillomycin L	(3)
Bacillus velezensis	T20E-257	Iturin	
Bacillus velezensis	TB1501	Iturin	
Bacillus velezensis	TH16	Bacillomycin L	
Bacillus velezensis	TJ02	Iturin	
Bacillus velezensis	TrigoCor1448	Iturin	
Bacillus velezensis	UASWS BA1	Iturin	
Bacillus velezensis	UASWSBA1	Iturin	
Bacillus velezensis	UBA5705	Bacillomycin D	
Bacillus velezensis	UCMB5033	Bacillomycin L	
Bacillus velezensis	UCMB5036	Iturin	
Bacillus velezensis	UCMB5113	Bacillomycin L	
Bacillus velezensis	UMAF6614	Bacillomycin D	
Bacillus velezensis	UMAF6639	Iturin	
Bacillus velezensis	UNC69MF	Bacillomycin L	
Bacillus velezensis	V4	Iturin	
Bacillus velezensis	VCC-2003	Iturin	

Table S2. Iturinic cluster assignment for all genomes analyzed.

Species	Isolate	Assignment	confirmed with MS/MS
Bacillus velezensis	W1	Bacillomycin D	
Bacillus velezensis	W2	Iturin	
Bacillus velezensis	WS-8	Iturin	
Bacillus velezensis	X1	Iturin	
Bacillus velezensis	XK-4-1	Iturin	
Bacillus velezensis	Y14	Iturin	
Bacillus velezensis	Y2	Bacillomycin D	
Bacillus velezensis	YJ11-1-4	Bacillomycin D	
Bacillus velezensis	ZeaDK315Endobac16	Bacillomycin D	
Bacillus velezensis	ZF2	Iturin	
Bacillus velezensis	ZL918	Iturin	

1. Lim, S. M., Yoon, M. Y., Choi, G. J., Choi, Y. H., Jang, K. S., Shin, T. S., Park, H. W., Yu, N. H., Kim, Y. H., and Kim, J. C. (2017) Plant Pathology Journal 33, 488-498.

2. Zhou, H., Luo, C., Fang, X., Xiang, Y., Wang, X., Zhang, R., and Chen, Z. C. (2016) PLoS ONE 11.

3. Li, X., Zhang, Y., Wei, Z., Guan, Z., Cai, Y., and Liao, X. (2016) PLoS ONE 11.

4. Luo, C., Zhou, H., Zou, J., Wang, X., Zhang, R., Xiang, Y., and Chen, Z. (2015) Applied Microbiology and Biotechnology 99, 1897-1910.

5. Pan, H., Tian, X., Shao, M., Xie, Y., Huang, H., Hu, J., and Ju, J. (2019) Applied Microbiology and Biotechnology 103, 4153-4165.

6. Luna-Bulbarela, A., Tinoco-Valencia, R., Corzo, G., Kazuma, K., Konno, K., Galindo, E., and Serrano-Carreón, L. (2018) Biological Control 127, 145-154.

7. Koumoutsi, A., Chen, X. H., Henne, A., Liesegang, H., Hitzeroth, G., Franke, P., Vater, J., and Borriss, R. (2004) Journal of Bacteriology 186, 1084-1096.

8. Yuan, J., Zhang, F., Wu, Y., Zhang, J., Raza, W., Shen, Q., and Huang, Q. (2014) Letters in Applied Microbiology 59, 169-176.

9. Pallazzini, J. M., Dunlap, C. A., Bowman, M. J., and Chulze, S. N. (2016) Microbiological Research 192, 30-36.

10. Dunlap, C. A., Bowman, M. J., and Schisler, D. A. (2013) Biological Control 64, 166-175.

11. Borriss, R., Chen, X. H., Rueckert, C., Blom, J., Becker, A., Baumgarth, B., Fan, B., Pukall, R., Schumann, P., Spröer, C., Junge, H., Vater, J., Pühler, A., and Klenk, H. P. (2011) International Journal of Systematic and Evolutionary Microbiology 61, 1786-1801.

12. Dang, Y., Zhao, F., Liu, X., Fan, X., Huang, R., Gao, W., Wang, S., and Yang, C. (2019) Microbial Cell Factories 18.

13. Fickers, P., Leclère, V., Guez, J. S., Béchet, M., Coucheney, F., Joris, B., and Jacques, P. (2008) Research in Microbiology 159, 449-457.

14. Knight, C., Bowman, M. J., Frederick, L., Day, A., Lee, C., and Dunlap, C. A. (2018) Microbiological Research, 40-46.

Table S3. Amino acid predictions based on NRPS substrate predictor[1]

	Adenylation domain			variable amino acids		
**Protein sample**	**start**	**finish**	**Score**	**predicted***	**observed**	**pcf error**
CBI43038.1_ItuB_iturin_(DSM_7)_amyloliquefaciens	3308	3717	800.6	Gln	Gln	0.0252
CBI43038.1_ItuB_iturin_(DSM_7)_amyloliquefaciens	4355	4754	659.4	Pro	Pro	0.0965
CBI43037.1_ItuC_iturin_(DSM_7)_amyloliquefaciens	289	687	929.1	Asn	Asn	0.0052
CBI43037.1_ItuC_iturin_(DSM_7)_amyloliquefaciens	1799	2209	817.3	Ser	Ser	0.0202
RDY88798.1_ItuB_bacillomycin_D_(CHCC26933)_amyloliquefaciens	3307	3707	573.9	Pro	Pro	0.1166
RDY88798.1_ItuB_bacillomycin_D_(CHCC26933)_amyloliquefaciens	4346	4755	673.4	Gln	Glu	0.0877
RDY88797.1_ItuC_bacillomycin_D_(CHCC26933)_amyloliquefaciens	288	699	821.6	Ser	Ser	0.0167
RDY88797.1_ItuC_bacillomycin_D_(CHCC26933)_amyloliquefaciens	1811	2213	754.1	Thr	Thr	0.0386
WP_088117316.1_ItuB_mycosubtilin_atrophaeus	3308	3717	786.8	Gln	Gln	0.0298
WP_088117316.1_ItuB_mycosubtilin_atrophaeus	4357	4756	667.6	Pro	Pro	0.0883
WP_088117315.1_ItuC_mycosubtilin_atrophaeus	288	698	851.6	Ser	Ser	0.0197
WP_088117315.1_ItuC_mycosubtilin_atrophaeus	1810	2208	864.3	Asn	Asn	0.0177
OLQ57028.1_ItuB_mojavensin_genomospecies	3308	3716	795.3	Gln	Gln	0.0281
OLQ57028.1_ItuB_mojavensin_genomospecies	4357	4750	643.1	Pro	Pro	0.105
OLQ57027.1_ItuC_mojavensin_genomospecies	289	687	902.6	Asn	Asn	0.013
OLQ57027.1_ItuC_mojavensin_genomospecies	1799	2197	877.9	Asn	Asn	0.0168
WP_069486605.1_ItuB_mojavensin_halotolerans	3307	3715	792	Gln	Gln	0.0278
WP_069486605.1_ItuB_mojavensin_halotolerans	4356	4749	650.7	Pro	Pro	0.1042
WP_069486604.1_ItuC_mojavensin_halotolerans	289	687	909.8	Asn	Asn	0.0036
WP_069486604.1_ItuC_mojavensin_halotolerans	1799	2197	880.3	Asn	Asn	0.0175
KXZ15117.1_ItuB_iturin_nakamurai	3309	3717	792.2	Gln	Gln	0.0278
KXZ15117.1_ItuB_iturin_nakamurai	4356	4755	647.5	Pro	Pro	0.1051
KXZ15116.1_ItuC_iturin_nakamurai	289	687	916.7	Asn	Asn	0.0041
KXZ15116.1_ItuC_iturin_nakamurai	1799	2208	725.4	Ser	Ser	0.0651

PAD65416.1_ItuB_iturin_(7551)_siamensis	3308	3716	840.6	Gln	Gln	0.0178
PAD65416.1_ItuB_iturin_(7551)_siamensis	4357	4750	667.3	Pro	Pro	0.0883
PAD65417.1_ItuC_iturin_(7551)_siamensis	289	687	924.4	Asn	Asn	0.0048
PAD65417.1_ItuC_iturin_(7551)_siamensis	1799	2209	828.9	Ser	Ser	0.0169
PIK31757.1_ItuB_bacillomycin_D_(scd15)_siamensis	3306	3706	568.7	Pro	Pro	0.1203
PIK31757.1_ItuB_bacillomycin_D_(scd15)_siamensis	4345	4754	665	Gln	Glu	0.0878
PIK31756.1_ItuC_bacillomycin_D_(scd15)_siamensis	288	698	824	Ser	Ser	0.0168
PIK31756.1_ItuC_bacillomycin_D_(scd15)_siamensis	1810	2212	761.4	Thr	Thr	0.0362
AMA52502.1_ituB_Bacillomycin_F_subtilis_subsp_inaquosorum	3309	3717	806.6	Gln	Gln	0.0194
AMA52502.1_ituB_Bacillomycin_F_subtilis_subsp_inaquosorum	4358	4757	657.8	Pro	Pro	0.0965
AMA52501.1_ituC_Bacillomycin_F_subtilis_subsp_inaquosorum	289	687	923.1	Asn	Asn	0.0048
AMA52501.1_ituC_Bacillomycin_F_subtilis_subsp_inaquosorum	1799	2201	764.2	Ser	Ser	0.0348
ADM37935.1_ItuB_mycosubtilin_subtilis_subsp_spizizeni	3310	3718	847.9	Gln	Gln	0.0187
ADM37935.1_ItuB_mycosubtilin_subtilis_subsp_spizizeni	4359	4758	665.8	Pro	Pro	0.0878
ADM37934.1_ItuC_mycosubtilin_subtilis_subsp_spizizeni	288	696	904.3	Ser	Ser	0.013
ADM37934.1_ItuC_mycosubtilin_subtilis_subsp_spizizeni	1808	2206	886.5	Asn	Asn	0.0151
OMI00189.1_ItuB_mojavensin_swezeyi	3311	3720	793.6	Gln	Gln	0.0278
OMI00189.1_ItuB_mojavensin_swezeyi	4360	4759	641.9	Pro	Pro	0.105
OMI00190.1_ItuC_mojavensin_swezeyi	289	687	923.5	Asn	Asn	0.0048
OMI00190.1_ItuC_mojavensin_swezeyi	1799	2197	881.3	Asn	Asn	0.0177
SPT99259.1_ItuB_mojavensin_tequilensis	3309	3717	839.8	Gln	Gln	0.0178
SPT99259.1_ItuB_mojavensin_tequilensis	4358	4757	666.7	Pro	Pro	0.0883
SPT99257.1_ItuC_mojavensin_tequilensis	289	687	934	Asn	Asn	0.0058
SPT99257.1_ItuC_mojavensin_tequilensis	1799	2197	873.1	Asn	Asn	0.0163
KSW05789.1_ItuB_bacillomycin_L_(KACC_18228)_velezensis	3309	3719	658	Ser	Ser	0.0965
KSW05789.1_ItuB_bacillomycin_L_(KACC_18228)_velezensis	4358	4767	666.1	Gln	Glu	0.0883
KSW05790.1_ItuC_bacillomycin_L_(KACC_18228)_velezensis	288	698	824.9	Ser	Ser	0.0168
KSW05790.1_ItuC_bacillomycin_L_(KACC_18228)_velezensis	1810	2212	751.1	Thr	Thr	0.0412

ABS74180.1_ItuB_bacillomycin_D_(FZB42)_velezensis	3307	3707	577.2	Pro	Pro	0.1135
ABS74180.1_ItuB_bacillomycin_D_(FZB42)_velezensis	4346	4755	672.7	Gln	Glu	0.0876
ABS74179.1_ItuC_bacillomycin_D_(FZB42)_velezensis	288	697	830.2	Ser	Ser	0.017
ABS74179.1_ItuC_bacillomycin_D_(FZB42)_velezensis	1810	2212	747.4	Thr	Thr	0.0409
AFZ90900.1_ItuB_iturin_(AS43.3)_velezensis	3309	3718	856.7	Gln	Gln	0.0215
AFZ90900.1_ItuB_iturin_(AS43.3)_velezensis	4358	4751	666.3	Pro	Pro	0.0883
AFZ90899.1_ItuC_iturin_(AS43.3)_velezensis	230	628	930.6	Asn	Asn	0.0054
AFZ90899.1_ItuC_iturin_(AS43.3)_velezensis	1740	2150	833.9	Ser	Ser	0.0175

*In all cases, the first three conserved amino acids were correctly predicted

1. NRPSsp: Non-Ribosomal Peptide Synthase substrate predictor
C Prieto; C Garcia-Estrada; D Lorenzana; JF Martin
Bioinformatics 2011; doi: 10.1093/bioinformatics/btr659

Figure S1. Mass spectroscopy assignments and reference data for all iturinic lipopeptides.

A.)

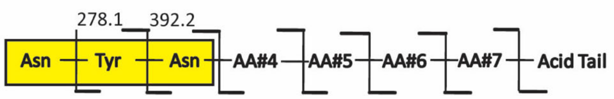

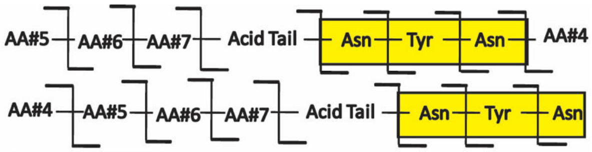

	chain length	[M+H]+	AA1	AA2	AA3	AA4	AA5	AA6	AA7			AA5	AA6	AA7		AA1	AA2	AA3	AA4
Iturin A	C14	1043.52	N	Y	N	Q	P	N	S	βAA	tail fragment	P	N	S	βAA	N	Y	N	Q
			**115.05**	**278.11**	**392.15**	**520.2**	**617.25**	**731.29**	**818.32**			**98.03**	**212.07**	**299.1**	524.33	638.37	801.43	915.47	
			1043.52	929.48	766.42	652.38	524.33	427.28	313.24	226.21	184.2		946.52	832.48	745.45	**520.22**	**406.18**	**243.12**	**129.08**
	C15	1057.54	N	Y	N	Q	P	N	S	βAA		P	N	S	βAA	N	Y	N	Q
			**115.05**	**278.11**	**392.15**	**520.20**	**617.25**	**731.29**	**818.32**			**98.03**	**212.07**	**299.10**	538.35	652.39	815.45	929.49	
			1057.54	943.50	780.44	666.40	538.35	441.30	327.26	240.23	198.22		960.54	846.50	759.47	**520.22**	**406.18**	**243.12**	**129.08**
	C16	1071.56	N	Y	N	Q	P	N	S	βAA		P	N	S	βAA	N	Y	N	Q
			**115.05**	**278.11**	**392.15**	**520.20**	**617.25**	**731.29**	**818.32**			**98.03**	**212.07**	**299.10**	552.37	666.41	829.47	943.51	
			1071.56	957.52	794.46	680.42	552.37	455.32	341.28	254.25	212.24		974.56	860.52	773.49	**520.22**	**406.18**	**243.12**	**129.08**
	C17	1085.58	N	Y	N	Q	P	N	S	βAA		P	N	S	βAA	N	Y	N	Q
			**115.05**	**278.11**	**392.15**	**520.20**	**617.25**	**731.29**	**818.32**			**98.03**	**212.07**	**299.1**	579.96	680.43	843.49	957.53	
			1085.58	971.54	808.48	694.44	566.39	469.34	355.30	268.27	226.26		988.58	874.54	787.51	**520.22**	**406.18**	**243.12**	**129.08**
			AA1	AA2	AA3	AA4	AA5	AA6	AA7			AA5	AA6	AA7		AA1	AA2	AA3	AA4
Mycosubtilin	C14	1043.52	N	Y	N	Q	P	S	N	βAA		P	S	N	βAA	N	Y	N	Q
			**115.05**	**278.11**	**392.15**	**520.20**	**617.25**	**704.28**	**818.32**			**98.03**	**185.06**	**299.1**	524.33	638.37	801.43	915.47	
			1043.52	929.48	766.42	652.38	524.33	427.28	340.25	226.21	184.2		946.52	859.49	745.45	**520.22**	**406.18**	**243.12**	**129.08**
	C15	1057.54	N	Y	N	Q	P	S	N	βAA		P	S	N	βAA	N	Y	N	Q
			**115.05**	**278.11**	**392.15**	**520.20**	**617.25**	**704.28**	**818.32**			**98.03**	**185.06**	**299.10**	538.35	652.39	815.45	929.49	
			1057.54	943.50	780.44	666.40	538.35	441.30	354.27	240.23	198.22		960.54	873.51	759.47	**520.22**	**406.18**	**243.12**	**129.08**
	C16	1071.56	N	Y	N	Q	P	S	N	βAA		P	S	N	βAA	N	Y	N	Q
			**115.05**	**278.11**	**392.15**	**520.20**	**617.25**	**704.28**	**818.32**			**98.03**	**185.06**	**299.10**	552.37	666.41	829.47	943.51	
			1071.56	957.52	794.46	680.42	552.37	455.32	368.29	254.25	212.24		974.56	887.53	773.49	**520.22**	**406.18**	**243.12**	**129.08**
	C17	1085.58	N	Y	N	Q	P	S	N	βAA		P	S	N	βAA	N	Y	N	Q
			**115.05**	**278.11**	**392.15**	**520.20**	**617.25**	**704.28**	**818.32**			**98.03**	**185.06**	**299.1**	579.96	680.43	843.49	957.53	
			1085.58	971.54	808.48	694.44	566.39	469.34	382.31	268.27	226.26		988.58	901.55	787.51	**520.22**	**406.18**	**243.12**	**129.08**
			AA1	AA2	AA3	AA4	AA5	AA6	AA7			AA4	AA5	AA6	AA7		AA1	AA2	AA3
Bacillomycin L	C14	1021.49	N	Y	N	S	E	S	T	βAA		S	E	S	T	βAA	N	Y	N
			**115.05**	**278.11**	**392.15**	**479.18**	**608.22**	**695.25**	**796.29**			**88.01**	**217.05**	**304.05**	**405.09**	630.32	744.36	907.42	1021.46
			1021.49	907.45	744.39	630.35	543.32	414.28	327.25	226.21	184.2	1021.52	934.49	805.45	718.42	617.38	**392.15**	**278.11**	**115.05**
	C15	1035.51	N	Y	N	S	E	S	T	βAA		S	E	S	T	βAA	N	Y	N
			**115.05**	**278.11**	**392.15**	**479.18**	**608.22**	**695.25**	**796.29**			**88.01**	**217.05**	**304.05**	**405.09**	644.34	758.38	921.44	1035.48
			1035.51	921.47	758.41	644.37	557.34	428.30	341.27	240.23	198.22	1035.56	948.51	819.47	732.44	631.40	**392.15**	**278.11**	**115.05**
	C16	1049.53	N	Y	N	S	E	S	T	βAA		S	E	S	T	βAA	N	Y	N
			**115.05**	**278.11**	**392.15**	**479.18**	**608.22**	**695.25**	**796.29**			**88.01**	**217.05**	**304.05**	**405.09**	658.36	772.40	935.46	1049.50
			1049.53	935.49	772.43	658.39	571.36	442.32	355.29	254.25	212.24	1049.58	962.53	833.49	746.46	645.42	**392.15**	**278.11**	**115.05**
	C17	1063.55	N	Y	N	S	E	S	T	βAA		S	E	S	T	βAA	N	Y	N
			**115.05**	**278.11**	**392.15**	**479.18**	**608.22**	**695.25**	**796.29**			**88.01**	**217.05**	**304.05**	**405.09**	672.38	786.42	949.48	1063.52
			1063.55	949.51	786.45	672.41	585.38	456.34	369.31	268.27	226.26	1063.6	976.55	847.51	760.48	659.44	**392.15**	**278.11**	**115.05**
			AA1	AA2	AA3	AA4	AA5	AA6	AA7			AA4	AA5	AA6	AA7		AA1	AA2	AA3
Bacillomycin D	C14	1031.51	N	Y	N	P	E	S	T	βAA		P	E	S	T	βAA	N	Y	N
			**115.05**	**278.11**	**392.15**	**489.20**	**618.24**	**705.27**	**806.31**			**98.03**	**227.07**	**314.1**	**415.14**	640.37	754.41	917.47	1031.51
			1031.51	917.47	754.41	640.37	543.32	414.28	327.25	226.21	184.2	1031.54	934.49	805.45	718.42	617.38	**392.15**	**278.11**	**115.05**
	C15	1045.53	N	Y	N	P	E	S	T	βAA		P	E	S	T	βAA	N	Y	N
			**115.05**	**278.11**	**392.15**	**489.20**	**618.24**	**705.27**	**806.31**			**98.03**	**227.07**	**314.10**	**415.14**	654.39	768.43	931.49	1045.53
			1045.53	931.49	768.43	654.39	557.34	428.30	341.27	240.23	198.22	1045.56	948.51	819.47	732.44	631.4	**392.15**	**278.11**	**115.05**
	C16	1059.55	N	Y	N	P	E	S	T	βAA		P	E	S	T	βAA	N	Y	N
			**115.05**	**278.11**	**392.15**	**489.20**	**618.24**	**705.27**	**806.31**			**98.03**	**227.07**	**314.10**	**415.14**	668.41	782.45	945.51	1059.55
			1059.55	945.51	782.45	668.41	571.36	442.32	355.29	254.25	212.24	1059.58	962.53	833.49	746.46	645.42	**392.15**	**278.11**	**115.05**
	C17	1073.57	N	Y	N	P	E	S	T	βAA		P	E	S	T	βAA	N	Y	N
			**115.05**	**278.11**	**392.15**	**489.20**	**618.24**	**705.27**	**806.31**			**98.03**	**227.07**	**314.1**	**415.14**	682.43	796.47	959.53	1073.57
			1073.57	959.53	796.47	682.43	585.38	456.34	369.31	268.27	226.26	1073.6	976.55	847.51	760.48	659.44	**392.15**	**278.11**	**115.05**
			AA1	AA2	AA3	AA4	AA5	AA6	AA7			AA5	AA6	AA7		AA1	AA2	AA3	AA4
Bacillomycin F	C14	1057.53	N	Y	N	Q	P	N	T	βAA		P	N	T	βAA	N	Y	N	Q
			**115.05**	**278.1**	**392.18**	**520.18**	**617.18**	**731.18**	**832.18**			**98.03**	**212.07**	**313.11**	538.34	652.38	815.44	929.48	
			1057.53	943.49	780.43	666.39	538.34	441.29	327.25	226.21	184.2		959.6	845.6	745.43	**520.2**	**406.2**	**243.12**	**129.08**
	C15	1071.55	N	Y	N	Q	P	N	T	βAA		P	N	T	βAA	N	Y	N	Q
			**115.05**	**278.10**	**392.18**	**520.18**	**617.18**	**731.18**	**832.18**			**98.03**	**212.07**	**313.11**	552.36	666.40	829.46	943.50	
			1071.55	957.51	794.45	680.41	552.36	455.31	341.27	240.23	198.22		973.62	859.62	759.45	**520.20**	**406.20**	**243.12**	**129.08**
	C16	1085.57	N	Y	N	Q	P	N	T	βAA		P	N	T	βAA	N	Y	N	Q
			**115.05**	**278.10**	**392.18**	**520.18**	**617.18**	**731.18**	**832.18**			**98.03**	**212.07**	**313.11**	566.38	680.42	843.48	957.52	
			1085.57	971.53	808.47	694.43	566.38	469.33	355.29	254.25	212.24		987.64	873.64	773.47	**520.20**	**406.20**	**243.12**	**129.08**
	C17	1099.59	N	Y	N	Q	P	N	T	βAA		P	N	T	βAA	N	Y	N	Q
			**115.05**	**278.10**	**392.18**	**520.18**	**617.18**	**731.18**	**832.18**			**98.03**	**212.07**	**313.11**	580.4	694.44	857.50	971.54	
			1099.59	985.55	822.49	708.45	580.40	483.35	369.31	268.27	226.26		1001.66	887.66	787.49	**520.20**	**406.20**	**243.12**	**129.08**
			AA1	AA2	AA3	AA4	AA5	AA6	AA7			AA5	AA6	AA7		AA1	AA2	AA3	AA4
Mojavensin A	C14	1070.53	N	Y	N	Q	P	N	N	βAA		P	N	N	βAA	N	Y	N	Q
			**115.05**	**278.11**	**392.15**	**520.20**	**617.25**	**731.29**	**845.33**			**98.03**	**212.07**	**326.11**	551.34	665.38	828.44	942.48	
			1070.53	956.49	793.43	679.39	551.34	454.29	340.25	226.21	184.2	1070.58	973.53	859.49	745.45	**520.22**	**406.18**	**243.12**	**129.08**
	C15	1084.55	N	Y	N	Q	P	N	N	βAA		P	N	N	βAA	N	Y	N	Q
			**115.05**	**278.11**	**392.15**	**520.20**	**617.25**	**731.29**	**845.33**			**98.03**	**212.07**	**326.11**	565.36	679.40	842.46	956.50	
			1084.55	970.51	807.45	693.41	565.36	468.31	354.27	240.23	198.22	1084.55	987.55	873.51	759.47	**520.22**	**406.18**	**243.12**	**129.08**
	C16	1098.57	N	Y	N	Q	P	N	N	βAA		P	N	N	βAA	N	Y	N	Q
			**115.05**	**278.11**	**392.15**	**520.20**	**617.25**	**731.29**	**845.33**			**98.03**	**212.07**	**326.11**	579.38	693.42	856.48	970.52	
			1098.57	984.53	821.47	707.43	579.38	482.33	368.29	254.25	212.24	1098.57	1001.57	887.53	773.49	**520.22**	**406.18**	**243.12**	**129.08**
	C17	1112.59	N	Y	N	Q	P	N	N	βAA		P	N	N	βAA	N	Y	N	Q
			**115.05**	**278.11**	**392.15**	**520.20**	**617.25**	**731.29**	**845.33**			**98.03**	**212.07**	**326.11**	593.40	707.44	870.50	984.54	
			1112.59	998.55	835.49	721.45	593.40	496.35	382.31	268.27	226.26	1112.59	1015.59	901.55	787.51	**520.22**	**406.18**	**243.12**	**129.08**

B.)

Iturin A

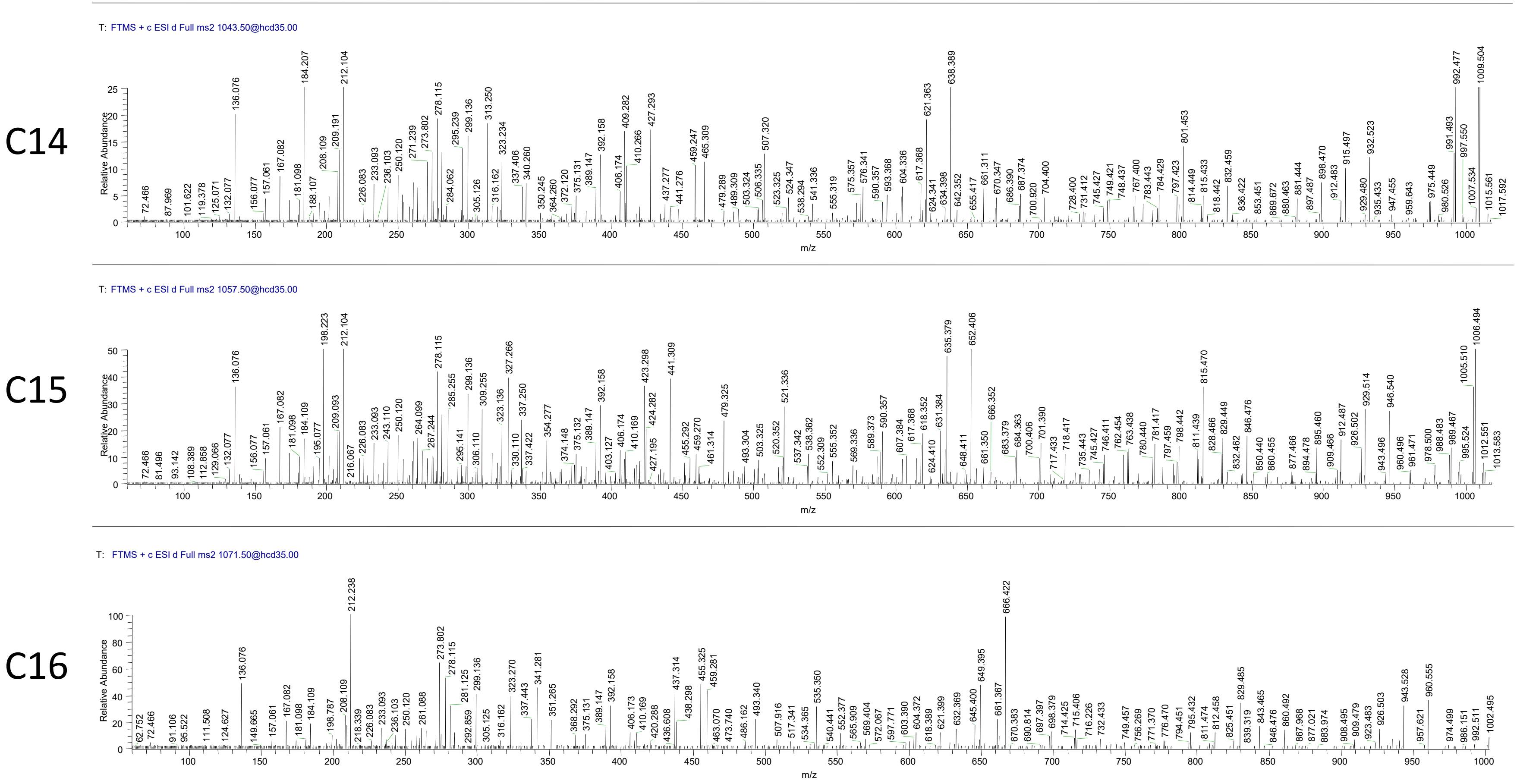
C14
T: FTMS + c ESI d Full ms2 1043.50@hcd35.00
Relative Abundance
m/z
C15
T: FTMS + c ESI d Full ms2 1057.50@hcd35.00
Relative Abundance
m/z
C16
T: FTMS + c ESI d Full ms2 1071.50@hcd35.00
Relative Abundance
m/z

C.)

Mycosubtilin

C15

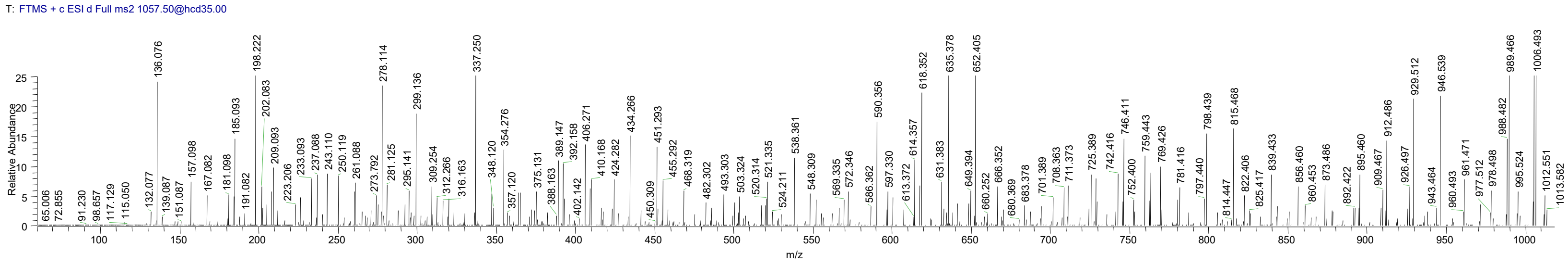

C16

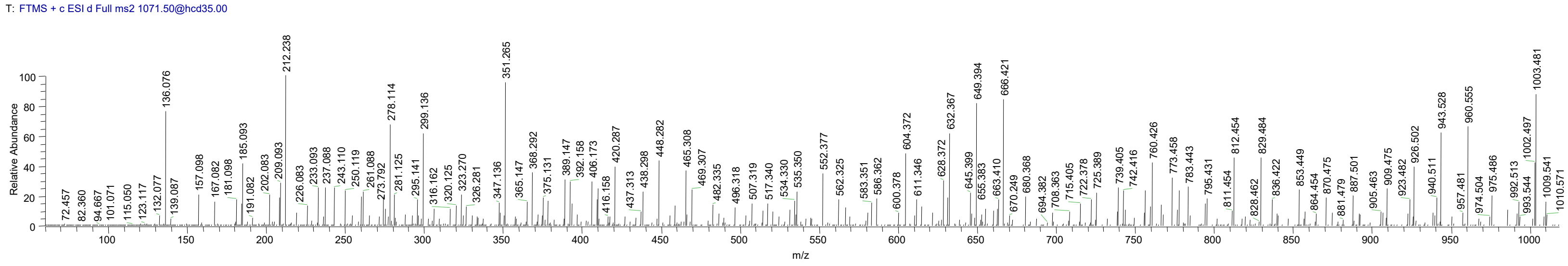

C17

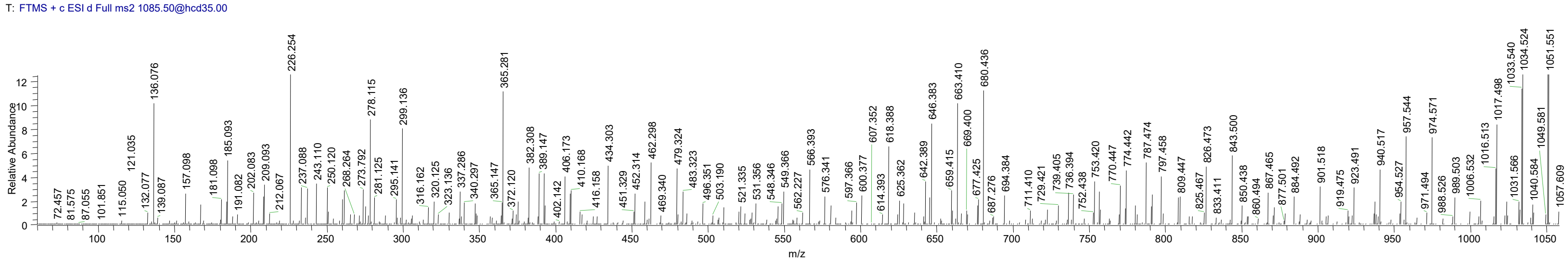

D.)

Bacillomycin L

C14

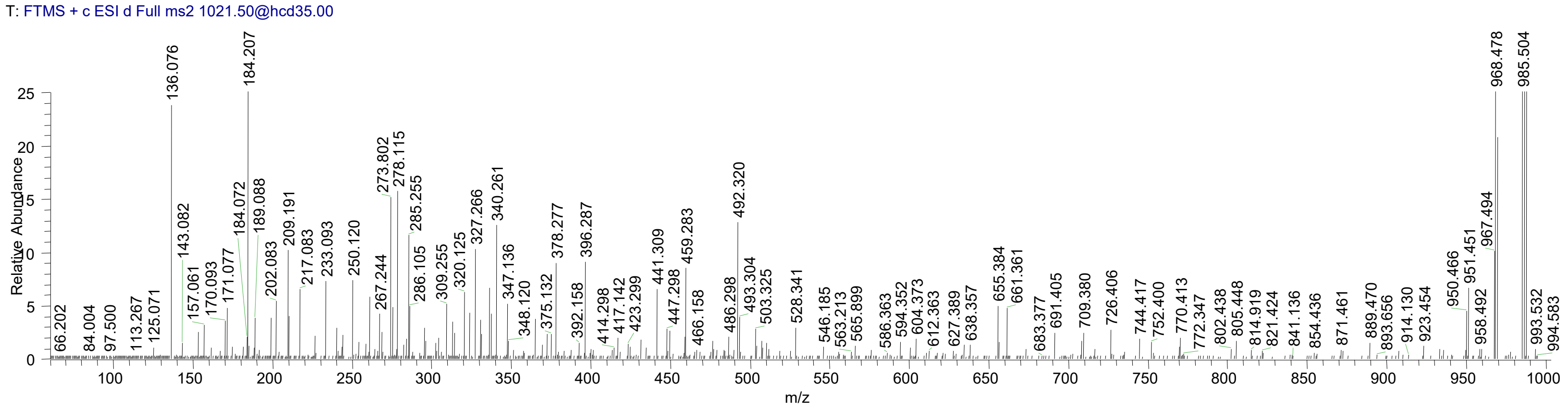

C15

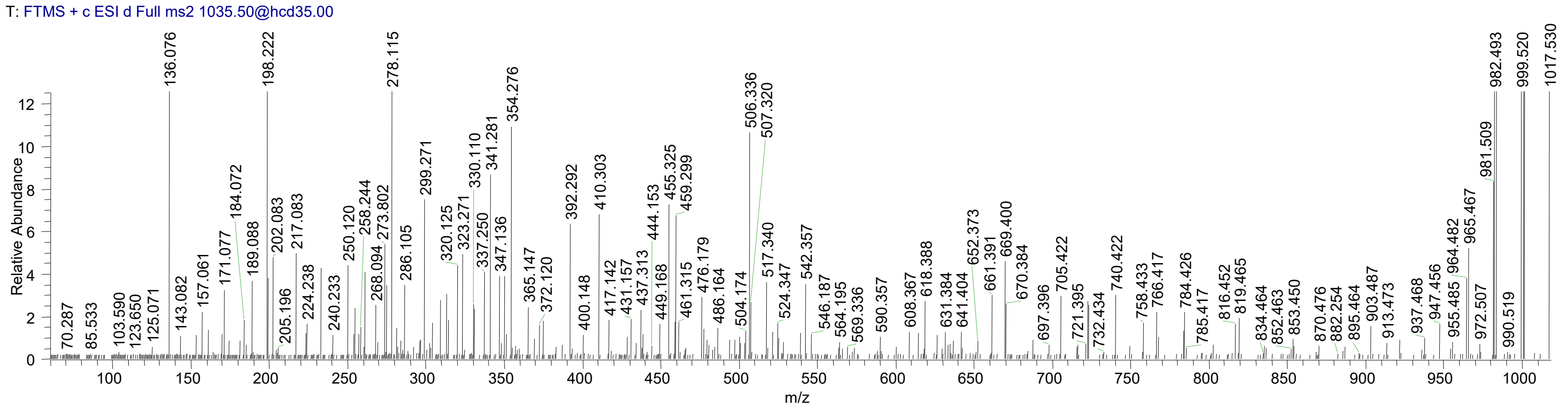

E.)

Bacillomycin D

C14

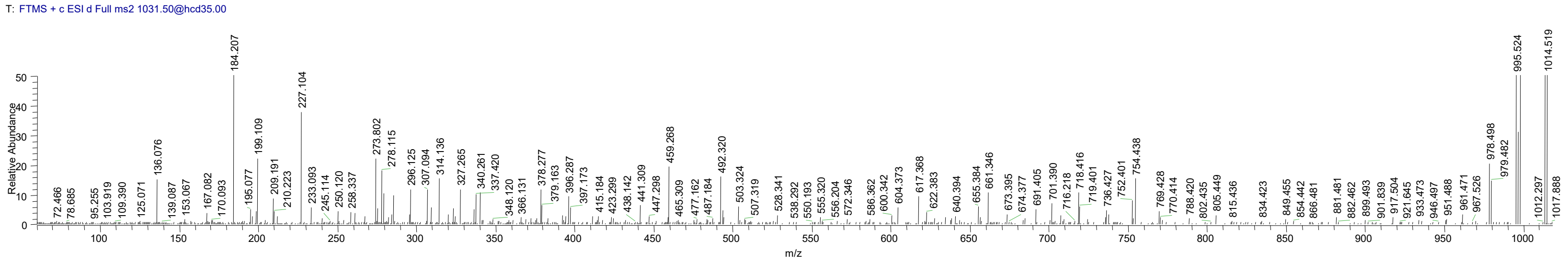

C15

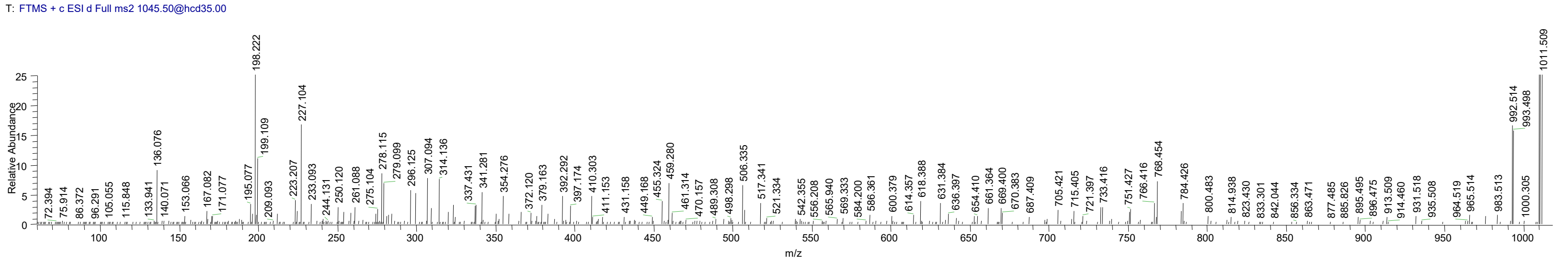

C16

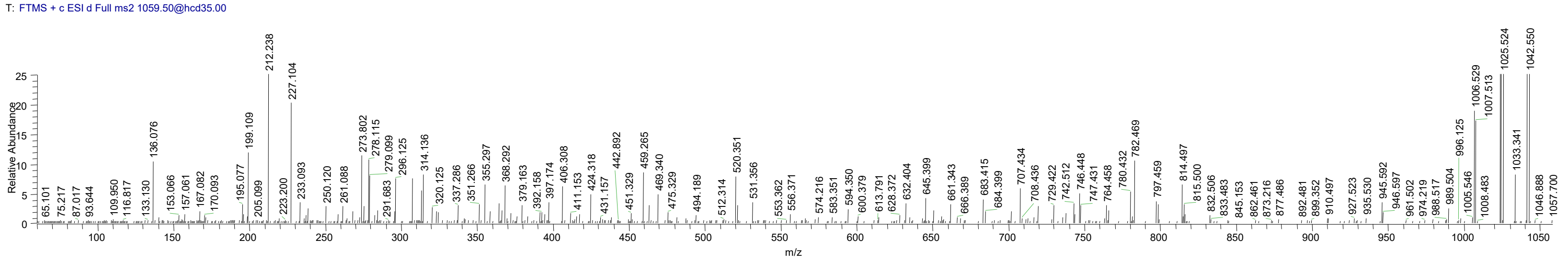

F.)

Bacillomycin F

C15

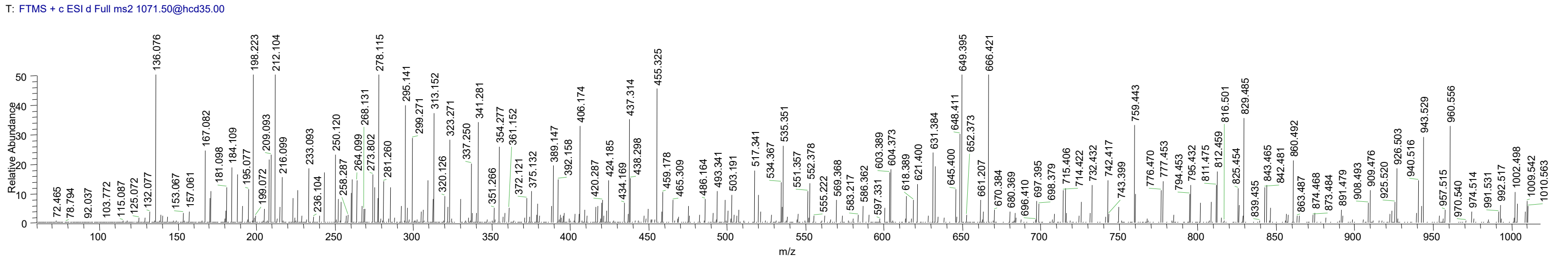

C16

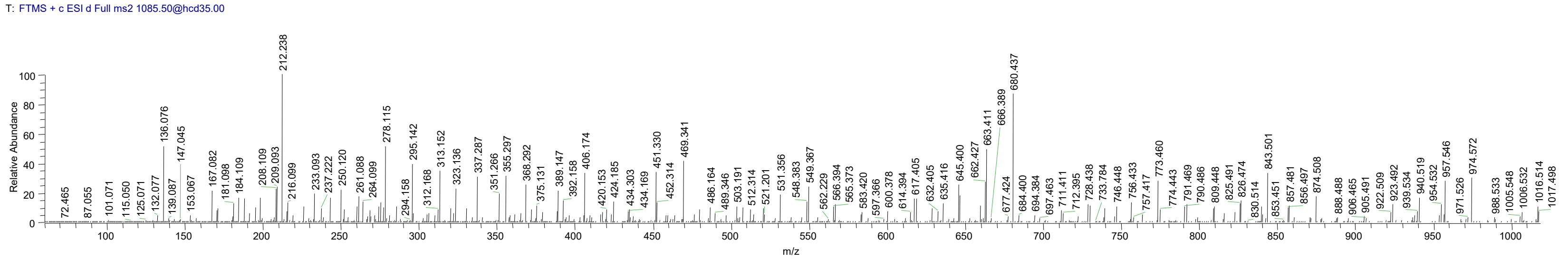

C17

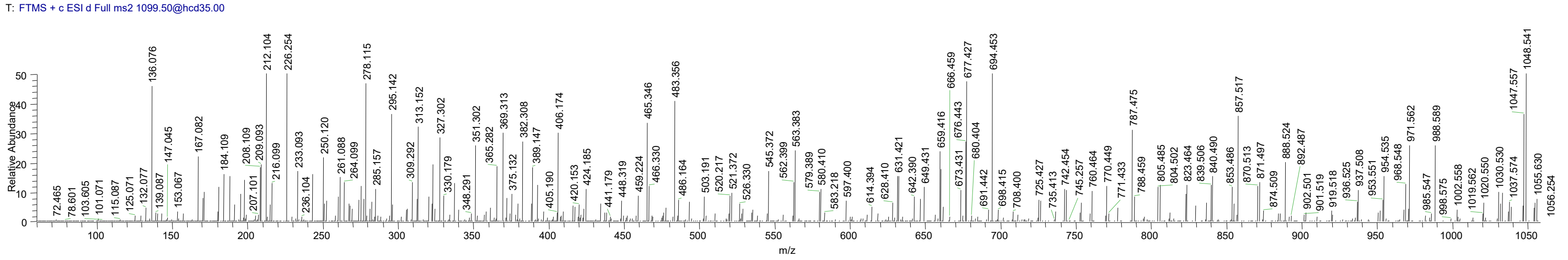

G.)

Mojavensin A

C15

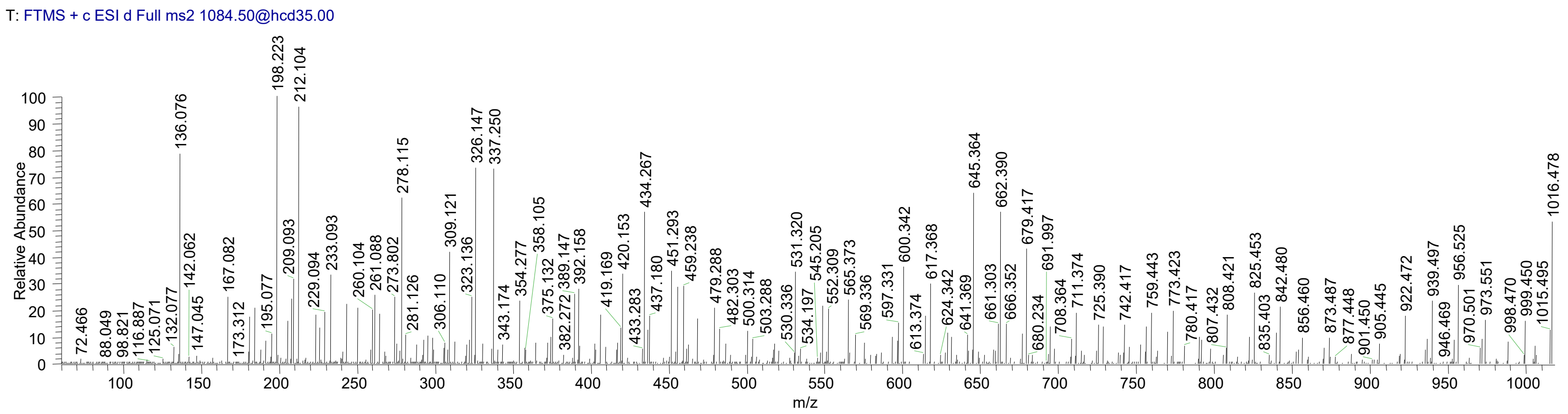

C13

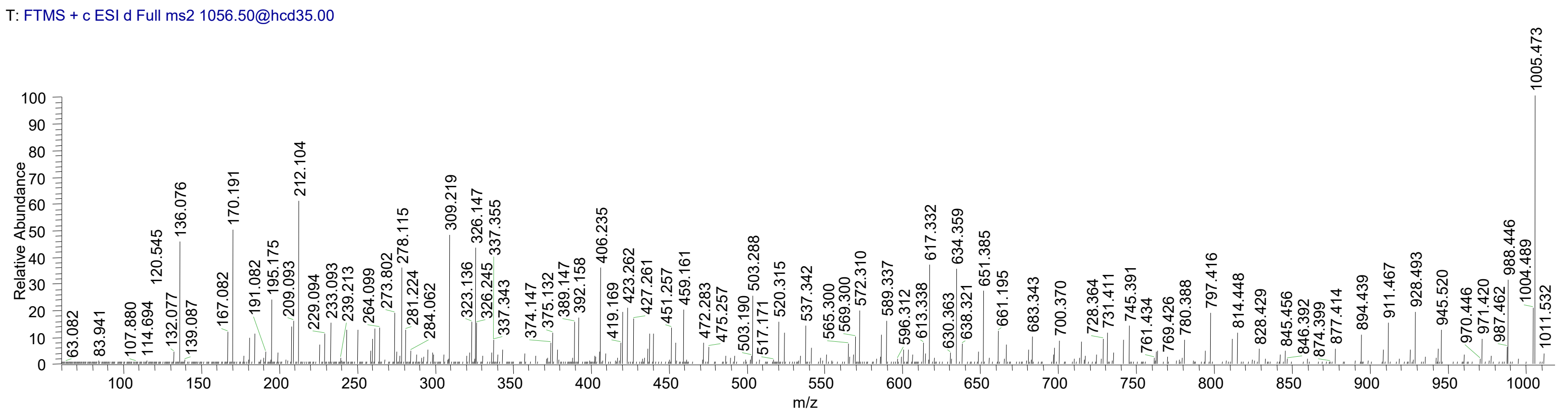

Supplement: Supplementary file 1 [file Data_Sheet_1.pdf]
